# Endophytic Bacterial Isolates From Halophytes Demonstrate Phytopathogen Biocontrol and Plant Growth Promotion Under High Salinity

**DOI:** 10.3389/fmicb.2021.681567

**Published:** 2021-05-04

**Authors:** Christos A. Christakis, Georgia Daskalogiannis, Anastasia Chatzaki, Emmanouil A. Markakis, Glykeria Mermigka, Angeliki Sagia, Giulio Flavio Rizzo, Vittoria Catara, Ilias Lagkouvardos, David J. Studholme, Panagiotis F. Sarris

**Affiliations:** ^1^Institute of Molecular Biology and Biotechnology, Foundation for Research and Technology – Hellas, Heraklion, Greece; ^2^Department of Biology, University of Crete, Heraklion, Greece; ^3^Laboratory of Mycology, Department of Viticulture, Vegetable Crops, Floriculture and Plant Protection, Institute of Olive Tree, Subtropical Crops and Viticulture, Hellenic Agricultural Organization DIMITRA, Heraklion, Greece; ^4^Department of Agriculture, Food and Environment, University of Catania, Catania, Italy; ^5^ZIEL–Institute for Food and Health, Technical University of Munich, Freising, Germany; ^6^Biosciences, University of Exeter, Exeter, United Kingdom

**Keywords:** halophytes, endophytes, stress tolerance, salinity tolerance, biofertilizers, biocontrol, bio-inoculants, growth-promotion

## Abstract

Halophytic endophytes potentially contribute to the host’s adaptation to adverse environments, improving its tolerance against various biotic and abiotic stresses. Here, we identified the culturable endophytic bacteria of three crop wild relative (CWR) halophytes: *Cakile maritima*, *Matthiola tricuspidata*, and *Crithmum maritimum*. In the present study, the potential of these isolates to improve crop adaptations to various stresses was investigated, using both *in vitro* and *in-planta* approaches. Endophytic isolates were identified by their 16S rRNA gene sequence and evaluated for their ability to: grow *in vitro* in high levels of NaCl; inhibit the growth of the economically important phytopathogens *Verticillium dahliae*, *Ralstonia solanacearum*, and *Clavibacter michiganensis* and the human pathogen *Aspergillus fumigatus;* provide salt tolerance *in-planta*; and provide growth promoting effect *in-planta*. Genomes of selected isolates were sequenced. In total, 115 endophytic isolates were identified. At least 16 isolates demonstrated growth under increased salinity, plant growth promotion and phytopathogen antagonistic activity. Three showed *in-planta* suppression of *Verticillium* growth. Furthermore, representatives of three novel species were identified: two *Pseudomonas* species and one *Arthrobacter*. This study provides proof-of-concept that the endophytes from CWR halophytes can be used as “bio-inoculants,” for the enhancement of growth and stress tolerance in crops, including the high-salinity stress.

## Introduction

Bacterial endophytes are widespread among plants and colonize intercellular and intracellular spaces of all host compartments. Each individual plant is a host to bacterial and fungal endophytes that colonize its tissues for all or part of its life cycle without causing any apparent pathogenesis (Ryan et al., 2008). Various studies have shown how microbial communities contribute to plant defense and the substantial beneficial effects they have on host plants, including improved nutrient acquisition, accelerated growth, resilience against pathogens and improved resistance against abiotic stress such as heat, drought, and salinity (Rodriguez et al., 2019).

The diversity and structure of endophytic microbiomes are dynamic and directly affected by ecological characteristics of the host plant and soil such as geographic location, environmental factors and interactions within the host plant (Edwards et al., 2015). Most characterized members of bacterial endophytic communities belong to the *Actinobacteria*, *Bacteroidetes*, *Firmicutes*, and *Proteobacteria* (Bulgarelli et al., 2013, 2015; Edwards et al., 2015). However, endophytic microbiome structure can be affected by the host-plant species’ genotype, plant organ or tissue type, developmental stage, growing season, geographic, and field conditions, soil type, nutrient status of the host species and cultivation practices (Rodríguez-Blanco et al., 2015; Liu et al., 2017; Rodriguez et al., 2019).

Endophytic microbes hold enormous potential to increase plant health. Interestingly, endophytic bacteria can be used to overcome the effect of salinity stress, promote plant growth and nutrient uptake; these approaches can provide beneficial and environmentally friendly solutions for a sustainable global food security (Glick, 2014; Tkacz and Poole, 2015; Vaishnav et al., 2019). For successful exploitation of endophytes, we need a deeper understanding of endophytic community composition and the mechanisms that underlie their plant growth promotion, in order to successfully select the most efficient bacterial isolates.

Members of endophytic bacterial communities influence each other with antagonistic, competitive, and mutualistic interactions (Toju et al., 2018). This results from nutritional competition, exchange and even metabolic interdependence. This, in turn can influence microbiome composition and its effect on the host-plant (Rodriguez et al., 2019). Host-plant genotype can also have a dramatic impact on microbial members; individual cultivars can influence the microbial community structure and the beneficial effects of endophytic bacteria (Haney et al., 2015; Marques et al., 2015; Pérez-Jaramillo et al., 2016; Rodriguez et al., 2019). Thus, for the utilization of endophytic bacterial isolates, an optimum approach is to isolate key bacterial strains from crop wild relatives (CWRs) (Mendes et al., 2013).

Halophytes could be valuable sources of novel endophytic isolates that can be used to overcome various biotic and abiotic stresses (Ruppel et al., 2013; Shabala, 2013; Yuan et al., 2016; Etesami and Beattie, 2018). High salinity in plants results in ionic and osmotic stress due increased extracellular hypertonic conditions and accumulation of Na^+^ and Cl^–^ intracellularly (Vaishnav et al., 2019). The resulting stress affects intracellular water balance, rate of cell division, hormonal imbalance, changes in photosynthesis, nutrient translocation, processes that decrease plant growth (Munns, 2002). Plant-associated microorganism can contribute to plant health impeded by salinity stress, by influencing phytohormonal levels and signaling, contributing to homeostasis maintenance of toxic ions under salinity stress, enhancing photosynthesis, and contributing to biomass production and allocation (Dodd and Pérez-Alfocea, 2012). Since soil salinity disrupts the physiological and morphological plant processes and increases pathogen susceptibility (Etesami and Beattie, 2018), the use of plant growth promoting endophytes in crops can be a more eco-friendly approach than agricultural chemicals.

Here, we tested the hypothesis that cultivated endophytic bacteria isolated from three CWR halophytic plant species have properties of salinity stress tolerance, plant growth promotion and phytopathogen growth inhibition. These species included two members of the *Brassicaceae* family (*Matthiola tricuspidata* and *Cakile maritima*), and one of the *Apiaceae* family (*Crithmum maritimum*). To test this hypothesis, we cultured and identified 115 different bacterial isolates and functionally characterized them in *in vitro* and *in-planta* assays. The bacterial isolates were tested *in vitro* for their ability to grow on salinity levels up to 17.5%, their biocontrol of the economically important plant phytopathogens *Verticillium dahliae*, *Ralstonia solanacearum*, and *Clavibacter michiganensis* ssp. *michiganensis.* Subsequently, isolates with demonstrated *in vitro* salt tolerance, were tested *in-planta* to demonstrate whether they promoted plant growth under no stress conditions and under high salinity. Furthermore, bacterial isolates were tested *in-planta* to check their biocontrol properties against *Verticillium dahliae*. This is the first study of bacterial endophytes obtained from *M. tricuspidata*, *Cr. maritimum*, and *Ca. maritima*, and identifies their potential as bacterial bio-inoculants in commercial crops to overcome salinity stress and plant diseases caused by the economically important pathogens.

## Materials and Methods

### Site Description and Plant Sample Collection

Samples were collected during summer 2018 in three distinct sites in Crete, Greece: site 1 (S1: N35°25′. E24°41′), site 2 (S2: N35°06, E25°48), and site 3 (S3: N35°00, E25°44) (Supplementary Figure 1). At S1, a natural beach area favoring salt-marsh vegetation, three *Matthiola tricuspidata* individuals were collected. At S2, a beach area close to Pachia Ammos village, three *Crithmum maritimum* individuals were collected. At S3, a popular beach area located in the town of Ierapetra, three individuals of *Cakile maritima* were collected. Each sample was collected with sterile gloves, forceps and gloves, placed in separate plastic bags to avoid cross contamination and immediately transported to the laboratory for processing.

### Plant Surface Sterilization and Endophytic Cell Isolation

Leaf and root materials from each species were cut and processed individually. Plant material was gently washed with sterile distilled water repeatedly to remove soil and dust particles. For surface sterilization, plant roots and leaves were placed into sterile Erlenmeyer flasks containing ethanol 75% v/v for 60 s with shaking and then in sterile Erlenmeyer flasks containing sodium hypochlorite solution 3% w/v^–1^ (NaClO) for 10 min. The plant materials were then placed again in ethanol 75% v/v for 60 s. To remove any remaining NaClO, plant materials were rinsed 10 times with sterile distilled water (dH_2_O). The sterilization and transfer procedures were carried out in a type II laminar flow hood. About 100 μL of the last rinse (for each analyzed sample) was plated on Nutrient Agar (NA) medium and monitored for microbial growth to evaluated surface sterilization efficiency. Only successfully sterilized root material was used further. Approximately 500 mg of leaves and roots per each species were weighed and slashed to small parts for further processing using a sterile scalpel and further grounded into a slurry with an autoclaved pestle and mortar. The slurry was transferred into sterile petri dishes and 30 mL of autoclaved dH_2_O was added. The petri dishes were sealed and placed onto a rotary shaker (150 rpm) at 25°C for 2 h. After shaking, 100 μl of the material in triplicate were inoculated on NA plates and incubated at 28°C. Colony forming units (cfus) were chosen from each plate based on their color, texture and morphology. Pure bacterial colonies were grown in Nutrient Broth (NB) and cells stocks were stored in 50% v/v glycerol at −80°C. A total of 115 isolates were identified.

### Bacterial Isolation and Identification of Isolates

To identify the 115 bacterial isolates, 16S rRNA gene Sanger sequencing method was employed. To extract crude genomic DNA, 1 mL of liquid bacterial culture in NB was placed in liquid nitrogen for 15 s. After room temperature incubation, the lysate was centrifuged at 10,000 × g for 1 min. Two microliter of the lysate were used to amplify the 16S rRNA gene using primers 27F: 5′-AGAGTTTGATCCTGGCTCAG-3′ (White et al., 1990) and 1492R: 5′-GTTTACCTTGTTACGACTT-3′ (Lane, 1991). PCR reactions of 20 μL were amplified in a BioRad T-100 Thermocycler with initial denaturation at 94°C for 2 min, followed by 35 cycles of 5 s at 94°C, 30 s annealing at 47°C, 2 min primer extension at 72°C, and a final extension at 72°C for 5 min. Apart from the lysate, each tube contained, Bac-Free PCR Buffer, 250 nM of each primer, 0.2 mM of each deoxy- ribonucleotide triphosphate and 0.1 U BAC-Free HotStart Taq polymerase (Nippon Genetics, Europe). PCR products were purified using Nucleo Spin Gel and PCR Clean up (Macherey-Nagel, Germany). Cleaned-up PCR products were sent to Macrogen (Europe) for sequencing with primer 27F.

The resulting chromatograms were quality inspected using MEGA 5 (Kumar et al., 2018) and the start/end regions of low quality were manually trimmed off. Cleaned-up fasta files were aligned in SILVA (Quast et al., 2013). The resulting sequences of the 16S rRNA gene were queried against ezBioCloud (Yoon et al., 2017) reference database for identification and documentation of the described bacterial isolate with the closest sequence similarity.

### Bacterial Salt Tolerance Assay

The salt tolerance of all bacterial isolates was estimated on the basis of the population density of these isolates at different concentrations of NaCl (ranging from 0.5, 5, 10, 15, and 17.5% (w/v) in NA. Ten microliter drops of freshly prepared NB cultures of each isolate were inoculated on sterilized petri plates, containing 25 mL NA with increasing NaCl concentrations and incubated at 28°C. For each NaCl concentration, an *Escherichia coli* laboratory isolate was inoculated as a negative control. After 24 h of incubation, the growth of each isolate was estimated compared to *E. coli* growth.

### *In vitro* Growth Inhibition of Phytopathogens

Antibacterial activity of the bacterial isolates against the phytopathogenic bacteria *Ralstonia solanacearum*, and *Clavibacter michiganensis* was evaluated by co-culturing each of the bacterial isolates on NA lawn covered by *R. solanacearum* or *C. michiganensis*. The inhibition zone indicating inhibition by bacterial growth was recorded as the antibacterial effect. Antifungal activity of the isolates against *Verticillium dahliae* was investigated. Potato dextrose agar (PDA) was inoculated with each bacterial isolate for 24 h at 28°C and then *V. dahliae* was inoculated at room temperature for 3–4 weeks. Fungal growth inhibition was determined by measuring the inhibition zone of *V. dahliae* hyphae on the media.

### *In vitro* Hemolysis Screening Assay

In order to assay the bacterial isolates for hemolytic activity, each isolate was grown on blood agar plates. The bacterial isolates were inoculated with the spot test method and were incubated at room temperature for 48 h. The known non-mammalian-pathogenic species *Ensifer meliloti* was employed as a negative control (Supplementary Figure 2B).

### *In vitro* Growth Inhibition of Fungal Human Pathogen

Antifungal activity of specific isolates against anthropopathogenic fungus *A spergillus fumigatus* was evaluated by co-culturing 11 bacterial isolates on NA plate lawn covered by *A. fumigatus* for 72 h at room temperature under absence of light. The following isolates were tested: CML04, CMR11, CMR22, CMR25, CrR12, CrR25, MTR12, MTR17a, MTR17b, MTR17c, MTR17d. Fungal growth inhibition was determined by the growth inhibition zone of the *A. fumigatus* hyphae on the media.

### *In-planta* Salt Tolerance Assays

Twelve of the bacterial isolates were selected, according to their ability to grow in high salinity conditions (up to 17.5% w/v NaCl), in order to test their plant growth promotion capacity of the model plant *Arabidopsis thaliana*. Firstly, the experiment was performed with no abiotic stress conditions. Bacterial isolates were cultured in NB for 46 h at 25°C with stirring. NB cultures were centrifuged at 224 × g for 15 min, the supernatant was discarded and the cells were resuspended in 50 mL sterilized dH_2_O. Seeds of *A. thaliana* ecotype Columbia (Col-0) were grown in plastic pots (6 × 6 × 7 cm) filled with vermiculite: soil (1:1), at 25°C (16 h light/8 h dark). For each isolate and the corresponding control, 5 individual plants were grown in each pot. *A. thaliana* plants were watered with dH_2_O for 10 days. Then, plants were watered with 10 mL suspensions of the 12 bacterial isolate liquid cultures, and were left for 7 days to let the bacterial isolates adapt. Subsequently, for a 30 day span, plants were watered every 2–3 days with dH_2_O. At the end of the experiment, the fresh weight of the leaves from each plant was measured. The leaves were then dried at 65–70°C for 2 days and their dry weight was measured.

The same experiment was performed under salt treatment. Specifically, after the 7 day period of bacterial isolate inoculation, instead of dH_2_O, the plants were watered with 10 mL of 250 mM NaCl. Fresh and dry weight of the leaves was measured.

For both experiments, mock samples were employed where no bacterial isolates were inoculated and control plants were inoculated with the isolate *Escherichia coli* (Control-*E. coli*), to check that the plants would not use the bacteria as a fertilizer.

### Confrontation and Volatile Tests of Selected Bacterial Isolates Against *Verticillium dahliae*

A total of 16 isolates were selected for direct *in vitro* antagonism of *V. dahliae*. Fourteen of these isolates were selected due to their strong inhibition of V. dahliae in initial tests (Supplementary Table 1; CrR14, CrR18, MTR18, CMR01, CMR03, CML04, CMR25, MTR17a, MTR17d, MTR17f, MTR17g, MTR17h, and MTR17b, MTR17c). Two additional isolates were selected (CrR04 and MTR12) with medium inhibition in initial tests (Supplementary Table 1) for comparison. Direct *in vitro* antagonism of *V. dahliae* was evaluated by dual-culture assays (confrontation test) on PDA (Lahlali et al., 2007). In particular, a 6 mm diameter mycelial disc taken from the periphery of a 2 week-old PDA fungal culture was placed on a new PDA plate (90 mm in diameter) at approximately 25 mm-distance from the center of the plate. Then, a 30 mm-long line from each bacterial isolate (taken from a 48 h-old tripticasein soy broth (TSB) liquid culture with an inoculation loop) was streaked on the opposite site of the plate at equal distance from the center (one isolate per plate). Moreover, *Trichoderma harzianum* strain T22 was isolated from the commercial biofungicide TRIANUM-P (Koppert B.V. Hellas) and included in *in vitro* bioassays for comparison. Plates inoculated only with *V. dahliae* agar discs were served as controls. Plates (three per bacterial isolate plus controls) were incubated at 24°C in the dark. The radius of fungal colonies toward the direction of the test isolate and that of controls was measured 5, 7, 9, and 12 days post inoculation (d.p.i.) and radial growth rates were expressed in mm/day. At the end of the bioassays (12 d.p.i.) the underside of the plates was scanned using a Samsung Xpress SL-M2875ND Laser Multifunction Printer at 1200 dpi and microsclerotial (black) area on each plate image was determined manually using the image processing software ImageJ 1.46r (National Institutes of Health, United States). Then, the number of spores was estimated by transferring a 6 mm-diameter disc taken from the periphery of each culture into a 1.5 mL Eppendorf tube with 1 mL of water, and vortexed for 30 s. The number of spores was measured using a haematocytometer under a light microscope. Moreover, actively growing mycelia from cultures’ periphery (located closer to test isolate) were prepared and microscopic observations (30 readings per culture) were carried out to estimate hyphae width.

To evaluate the capacity of bacterial isolates to affect *V. dahliae* growth via the production of volatile compounds, dual-plate assays (Chaurasia et al., 2005) were conducted (volatile test). In brief, one 6 mm-diameter agar disc of actively growing mycelium of the fungus was placed in the center of a new PDA plate (90 mm in diameter), whilst each bacterial isolate (taken from a 48 h-old TSB liquid culture) was streaked on another PDA plate. The covers of the two plates were removed and resultant plates were adjusted together (bacterial culture was upturned) and sealed with cellophane membrane so the two microbes would share the same headspace without coming in contact with each other. Dual plates (upright and upturned) inoculated only with *V. dahliae* served as controls. Similarly, in dual-culture assays, dual-plates (three per bacterial isolate) were incubated at 24°C in the dark and the growth, microclerotial area, sporulation and hyphae width of fungal colonies were measured as described above.

Radial growth inhibition (RGI), microsclerotia formation inhibition (MFI), sporulation inhibition (SI) and hyphae thinning (HT) were calculated according to the formula: [(Vc-Vt)/Vc] × 100 where Vc = the microscopic value of *V. dahliae* in control plates and Vt = the respective value of *V. dahliae* against the antagonistic isolate in dual-culture or dual-plate assays.

### Bacterial Isolates and Fungal Inoculum Preparation for *in-planta* Bioassays

The 16 selected (see above) bacterial isolates (CrR14, CrR18, CrR04, MTR12, MTR18, CMR01, CMR03, CML04, CMR25, MTR17a, MTR17d, MTR17f, MTR17g, MTR17h, and MTR17b, MTR17c) were used in *in-planta* bioassays. The isolates were grown in Erlenmeyer flasks with 200 mL liquid TSB, in an orbital incubator at 180 rpm and 28°C for 48 h in the dark. Bacterial suspensions were centrifuged at 3,000 × g for 10 min and cells were re-suspended in water reaching a final concentration of 10^8^ cfu mL^–1^ (measured by dilution plating).

The highly virulent *V. dahliae* isolate 999-1 (Markakis et al., 2016), which originated from symptomatic eggplants (*Solanum melongena* L.), was used. *V. dahliae* conidial suspension for eggplant—*V. dahliae* bioassays was prepared as previously described (Markakis et al., 2016). In brief, conidia were produced by growing each *V. dahliae* strain in potato dextrose broth (PDB) at 160 rpm and 25°C in the dark for 5 days. Then, conidia were harvested by filtrating through three layers of cheesecloth and the suspensions centrifuged at 3,000 × g for 10 min. Spores were re-suspended in sterilized dH_2_O and their concentration was adjusted to 5 × 10^6^ conidia mL^–1^.

### *In-planta* Verticillium Wilt Suppression Bioassays

Eggplant seedlings (cv. Black Beauty) were used in the *in-planta* bioassays. Plants at the one-true-leaf stage, grown in 100 mL-capacity pots containing soil substrate (HuminSubstrat, Klasmann-3 Deilmann GmbH, Germany) were root-drenched with bacterial suspension (20 mL of 10^8^ cfu mL^–1^ of each isolate per plant), whereas plants that served as controls (negative = no bacterium/no fungus assigned as “C−” and positive = no bacterium/plus pathogen assigned as “V.D.”) were treated with 20 mL of water. One week later, eggplants (at the second-true-leaf stage) were inoculated with *V. dahliae* by drenching the soil substrate in each pot with conidial suspension (20 mL of 5 × 10^6^ conidia mL^–1^ per pot). Negative control plants (C−) were treated with 20 mL of water. Eggplants were maintained under controlled conditions at 23 ± 2°C with a 12 h light and dark cycle.

Two independent experiments (experiments I and II ) were conducted to evaluate the suppressive effect of the aforementioned bacterial isolates against *V. dahliae*. In experiment I, 11 treatments (C−, V.d., V.d. + CrR14, V.d. + CrR18, V.d. + CrR04, V.d. + MTR12, V.d. + MTR18, V.d. + CMR01, V.d. + CMR03, V.d. + CML04 and V.d. + CMR25) were applied; whereas in experiment II, 10 treatments were conducted (C−, V.d., V.d. + MTR17a, V.d. + MTR17d, V.d. + BMTR17f, V.d. + MTR17g, V.d. + MTR17h, V.d. + MTR17b, V.d. + MTR17c, and V.d. + TRIANUM-P). The commercial biofungicide TRIANUM-P was included in experiment II (assigned as V.d. + TRIANUM-P) and applied according to manufacturer’s instruction (20 mL of 3 × 10^7^ cfu mL^–1^ per plant). TRIANUM-P was served as a *V. dahliae*-suppressive reference treatment. Within each experiment, each treatment consisted of seven plants and experiments were replicated three times.

### Disease Assessment

Verticillium wilt symptoms on eggplant were recorded at 2-, 3-, and 4- day intervals from 12 to 30 d.p.i with *V. dahliae*. Bioassays were evaluated by estimating disease severity, disease incidence, mortality and relative area under disease progress curve (RAUDPC). Disease parameters were recorded as previously described (Markakis et al., 2016). Briefly, disease severity at each observation was calculated from the number of wilting leaves, as a percentage of total number of leaves per each plant. Disease ratings were plotted over time to generate disease progress curves. Subsequently the area under disease progress curve (AUDPC) was calculated by the trapezoidal integration method (Campbell and Madden, 1990). Disease was expressed as a percentage of the maximum possible area with reference to the maximum value potential reached over the whole period of each experiment and is referred to as RAUDPC. Disease incidence was estimated as the percentage of infected plants. Only plants with a final disease severity of ≥20% were considered infected, to discriminate between *V. dahliae*-associated disease symptoms and other weak symptoms occasionally observed (Supplementary Table 2). Mortality was estimated as the percentage of dead plants.

### Plant Growth

Growth parameters were evaluated at the end of bioassays (at 24 and 30 d.p.i. for experiments I and II, respectively). To estimate the effect of the aforementioned treatments on plant growth, all plants were clipped off at the soil surface level and their height, fresh weight and leaf number were measured.

### Fungal Pathogen Re-isolation

To verify the presence of the applied *V. dahliae* strain in plant tissues, five plants per treatment in each experiment were randomly selected. Eggplant leaves which had been cut above soil level previously were removed and their stems were surface-disinfected by spraying with 95% ethyl alcohol and by quickly passing them through flame three times. For each plant, 3 xylem chips taken from different sites along the stem (base, middle and upper part of the stem) and aseptically placed onto acidified PDA after removing the phloem. Plates were then incubated at 24°C in the dark for 14 days. The emerging fungi that grew out of tissue excisions were examined visually and under a light microscope and identified according to their morphological characteristics (Pegg and Brady, 2002). Pathogen isolation ratio was expressed as the frequency of positive *V. dahliae* isolations of each plant.

### Statistics

Analysis of variance (ANOVA) was employed to determine the effects of replication (1, 2, or 3), treatment (C−, V.d., V.d. + CrR14, V.d. + CrR18, V.d. + CrR04, V.d. + MTR12, V.d. + MTR18, V.d. + CMR01, V.d. + CMR03, V.d. + CML04, V.d. + CMR25 in Experiment I and C−, V.d., V.d. + MTR17a, V.d. + MTR17d, V.d. + BMTR17f, V.d. + MTR17g, V.d. + MTR17h, V.d. + MTR17b, V.d. + MTR17c, V.d. + TRIANUM-P in experiment II ) and their interaction on disease incidence (DI), final disease severity (FDS), mortality (M), RAUDPC and isolation ratio (IR), and on plant height, fresh weight and total number of leaves (Supplementary Tables 2, 3). Prior to ANOVA, normality of data and homogeneity of variance across treatments was evaluated and an arcsine transformation was applied to normalize variance. When a significant *F* test was obtained for treatments (*P* ≤ 0.05), the data were subjected to means separation by Tukey’s honestly significant difference test. Morphological and physiological characteristics of *V. dahliae* in dual-culture and dual-plate assays were also analyzed by Tukey’s test (*P* ≤ *0.05*). Moreover, standard errors of means were calculated.

### Bacterial Genome Sequencing and Annotation

Twelve bacterial isolates were selected for whole genome sequencing (CrR16, CMR16, CrR07, CMR13, CrR06, CrR18, CrR14, CMR27, CMR25, CML04, CMR29, CrR25). The isolates were selected when they met more than two of the following criteria: (a) the 16S rRNA gene sequence of the isolates being 99.6% similar to their closest relative or lower, (b) exhibiting salt tolerance higher than the 5% threshold, (c) exhibiting medium or strong inhibition against the growth of at least 2 of the 3 tested phytopathogens *Verticillium dahliae*, *Ralstonia solanacearum*, and *Clavibacter michiganensis* ssp. *michiganensis* (Supplementary Table 1). For each isolate a 250 bp paired-end library was produced for use with the Illumina MiSeq sequencing system (University of Exeter Sequencing Service, Exeter, United Kingdom). Reads were assembled using SPAdes 3.12.0 (Bankevich et al., 2012) and the assembled sequence was annotated using the NCBI Prokaryotic Genome Annotation Pipeline (PGAP). Raw sequence reads and assembled genomes were uploaded to the Sequence Read Archive (Leinonen et al., 2011) and GenBank (Dennis Benson et al., 2017) and are available under BioProject accession number PRJNA634334. RAST (Rapid Annotation using Subsystem Technology) (Overbeek et al., 2014) was employed for genome analysis and annotation.

### Sequence Alignment and Phylogenetic Tree Construction

*S*elected gene sequences were aligned with ClustalX v2.0 (Larkin et al., 2007) and subsequently manually corrected. Sequence relationships were inferred using the maximum-likelihood (ML) method. ML phylogenies were constructed using MEGA 5.2 (Tamura et al., 2011). Phylogenetic trees were constructed using the concatenated *recA* and *gyrB* genes and assuming the bootstrap value derived from 1,500 replicates to represent the evolutionary history of the included taxa.

The evolutionary history of *Arthrobacter recA-gyrB* genes were inferred by using the Maximum Likelihood method based on the Tamura-Nei model (Tamura and Nei, 1993). The bootstrap consensus tree inferred from 500 replicates (Felsenstein, 1985) was taken to represent the evolutionary history of the taxa analyzed (Felsenstein, 1985). Branches corresponding to partitions reproduced in less than 50% bootstrap replicates are collapsed. Initial tree(s) for the heuristic search were obtained automatically as follows. When the number of common sites was <100 or less than one fourth of the total number of sites, the maximum parsimony method was used; otherwise BIONJ method with MCL distance matrix was used. Trees were drawn to scale, with branch lengths measured in the number of substitutions per site. The analysis involved 25 nucleotide sequences. Codon positions included were 1st + 2nd + 3rd + Non-coding. All positions with less than 95% site coverage were eliminated. That is, fewer than 5% alignment gaps, missing data, and ambiguous bases were allowed at any position. Evolutionary analyses were conducted in MEGA5 (Tamura et al., 2011).

The evolutionary history *Arthrobacter recA-gyrB* genes was inferred with the Maximum Likelihood method as above. The tree with the highest log likelihood (−7831.6808) was selected. Initial tree(s) for the heuristic search were obtained automatically as follows. When the number of common sites was <100 or less than one fourth of the total number of sites, the maximum parsimony method was used; otherwise BIONJ method with MCL distance matrix was used. The bootstrap consensus tree inferred from 500 replicates (Felsenstein, 1985) is taken to represent the evolutionary history of the taxa analyzed (Felsenstein, 1985). The tree was drawn to scale, with branch lengths measured in the number of substitutions per site. The analysis involved 22 nucleotide sequences. Codon positions included were 1st + 2nd + 3rd + Non-coding. All positions with less than 95% site coverage were eliminated as described above. Evolutionary analyses conducted in MEGA5 as described above.

## Results

### Identification and Abundance of Culturable Endophytic Bacteria

Endophytic bacteria were cultivated from different surface-sterilized tissue samples from all three halophytes. A total of 115 pure bacterial cultures showing different colony morphology (from root or leaf) were obtained; 91 were retrieved from roots and 24 from leaves. In detail, 45, 31, and 39 isolates were obtained from *M. tricuspidate, Ca. maritima*, and *Cr. Maritimum*, respectively (Supplementary Table 1).

**FIGURE 1:**
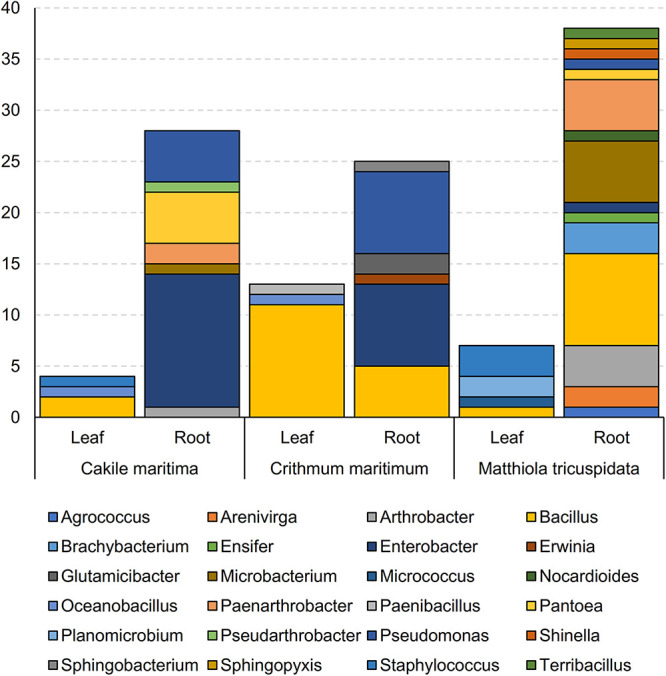
Abundance of genera of bacterial isolates obtained from leaves and roots of *Matthiola tricuspidata*, *Crithmum maritimuma*, and *Cakile maritima* plants.

For all 115 isolates, total 16S rRNA gene sequencing allowed for taxonomic analysis (Figure 1 and Supplementary Table 1). Bacterial isolates were assigned to 5 different classes (Supplementary Table 1) and 24 genera (Figure 1 and Supplementary Table 1). The most prevalent genus was *Bacillus*, accounting for 24% of the isolates, followed by *Enterobacter* (19%) and *Pseudomonas* (12%). The highest number of bacteria were isolated from roots of *M. tricuspidata* (38), followed by *Ca. maritima* roots (28) and *Cr. maritimum* roots (25), in contrast to the number of bacteria isolated from leaf samples (*M. tricuspidata*: 7, *Ca. maritima*: 4 and *Cr. maritimum*: 13). Isolates of genus *Bacillus* were isolated from all plants and tissues except the roots of *Cr. maritimum*, while *Pseudomonas* isolates were only isolated from root samples.

Apart from *Bacillus*, which was isolated from both roots and leaves, the rest of the genera were isolated only from roots or leaves (Supplementary Table 1). Similarly, all isolates from the genera *Enterobacter, Pseudomonas, Microbacterium, Paenarthrobacter, Pantoea, Arthrobacter, Brachybacterium, Arenivirga*, and *Glutamicibacter* were isolated from root samples, whereas the isolates *Oceanobacillus, Planomicrobium*, and *Staphylococcus* were isolated only from leaf samples (Supplementary Table 1).

The most frequently isolated genera were hosted in at least two of the three halophyte species. *M. tricuspidata* hosted the largest number of genera (18). *Ca. maritima* hosted the largest number of *Enterobacter* (13 out of 22) and the smallest number of *Bacillus* (2 out of 28). Members of the genera *Brachybacterium* and *Arenivirga* were isolated only from *M. tricuspidata* plants, whilst *Glutamicibacter* were isolated only from *Cr. maritimum* and the *Planomicrobium* from *Ca. maritima.*

### Bacterial Growth Under Salinity Stress

Bacterial isolates were tested for their ability to grow in elevated NaCl concentrations (5, 7.5, 10, 15, and 17.5%) (Supplementary Figure 2A). Most isolates showed growth at 5% NaCl (96 isolates). From the 28 isolates from *Ca. maritima* roots, 26 isolates (92.9% of the total) showed ability to grow at 5% salinity, and 14 of these (50%) showed growth at 7.5% salinity; all four isolates from the leaves of the same plant showed growth at 10% salinity and two of these could grow at 17.5%. 21 out of 25 isolates (84%) of the roots of *Cr. maritimum* could grow at 5% salt and 16 (64%) could grow at 10% salinity. 12 out of 13 (92.3%) isolates from the leaves of *Cr. maritimum* could grow at the 5% level and eight (61.5%) could grow at 10% salt. From the 38 isolates obtained from the root of *M. triscupidata*, 27 (71%) could grow at the 5% salt threshold and three could grow in 10% salt.

Of the six isolates that managed to grow at 17.5% salinity, four were isolated from leaf tissues (Supplementary Table 1): *Staphylococcus saprophyticus* (CML12) and *Oceanobacillus picturae* (CML15) isolated from *Ca. maritima* leaves, *Oceanobacillus picturae* (CrL11) from *Cr. maritimum* leaves, and *Micrococcus aloeverae* (MTL04) from *M. triscupidata* leaves (Supplementary Table 1 and Supplementary Figure 2A). The two isolates from root tissues that could grow on 17.5% are *Enterobacter hormaechei* subsp. *hoffmannii* (CMR13) isolated from *Ca. maritima* and *Bacillus hwajinpoensis* (CrR23) isolated from *Cr. maritimum* (Supplementary Table 1 and Supplementary Figure 2A). Another three *Bacilli* isolates (CrR16: *Bacillus haikouensis*, CrR22: *Bacillus haikouensis*, MTR05: *Terribacillus saccharophilus*) showed growth on 15% salinity (Supplementary Table 1 and Supplementary Figure 2A).

### Phytopathogens Growth Inhibition Ability

**FIGURE 2:**
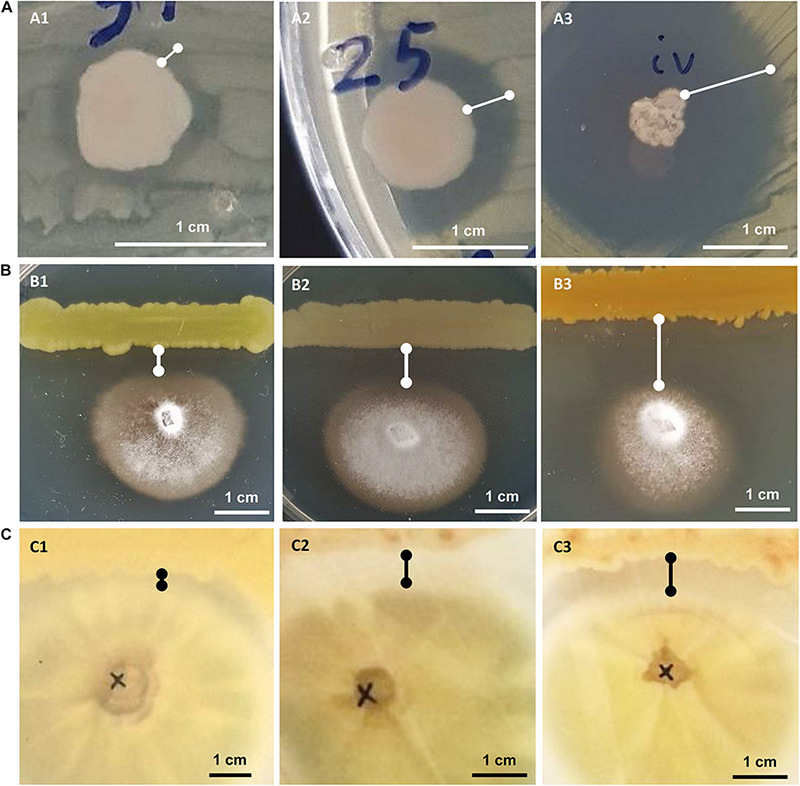
*In vitro* growth inhibition experiments: against phytopathogenic bacteria *Ralstonia solanacearum* or *Clavibacter michiganensis* **(A)**, against phytopathogenic fungus *Verticillium dahliae* **(B)** and against human pathogenic fungus *Aspergillus fumigatus* **(C)**. Representative experiments are shown to display their “weak” (A1–C1), “medium” (A2–C2), or “strong” (A3–C3) inhibitory activity.

All bacterial isolates were subjected to *in vitro* inhibition assays against three known phytopathogens: the bacteria *Ralstonia solanacearum* and *Clavibacter michiganensis* subsp. *michiganensis* and the fungus *Verticillium dahliae*. In the assays against the phytopathogenic bacteria, the bacterial isolates that showed any kind of inhibition were characterized as having “weak,” “medium” or “strong” inhibitory activity based on the size of the inhibition zone around the bacterial colony (Figure 2). In the *in vitro* assay against *Verticillium* the inhibitory activity was similarly judged as “weak,” “medium” or “strong” based on the linear distance between the bacterial and the fungal colonies (Figure 2).

Twenty-five (21.7%) out of 115 bacterial isolates demonstrated inhibition of the *Ralstonia solanacearum* growth (Supplementary Table 1). These 25 isolates belong to six genera: *Bacillus, Enterobacter, Erwinia, Glutamicibacter, Paenarthrobacter*, and *Pseudomonas*. Isolate CML04 (*Bacillus altitudinis*), obtained from leaf tissues of *Ca. maritima*, was the only leaf-derived isolate that showed antagonistic activity against all 3 tested phytopathogens (Supplementary Table 1). Of the 45 isolates isolated from *M. triscupidata*, three isolates showed a weak inhibitory zone against *Ralstonia*. A total of 10 isolates belonging to the genera *Enterobacter* and *Pseudomonas* (Supplementary Table 1) showed a strong inhibition (Figure 2).

A lower number of isolates showed any kind of inhibition against the phytopathogenic *Clavibacter michiganensis* subsp. *michiganensis*: 17 isolates out of 115 (14.8%). All 9 isolates from *M. triscupidata* (*Bacillus licheniformis* or *Bacillus sonorensis* isolates) demonstrated a strong inhibition zone (Figure 2). Similarly, two isolates from *Cr. maritimum* roots (both *Pseudomonas glareae*, CrR12 and CrR13) showed a strong inhibition zone (Supplementary Table 1). An additional two and four isolates showed weak and medium inhibition zone against *Clavibacter*, respectively (Supplementary Table 1).

The majority (76.5%) of the bacterial isolates demonstrated inhibition of the phytopathogenic fungus *Verticillium dahliae* (Supplementary Table 1). These isolates originate from both leaf and root tissues from all three halophytes. A strong inhibition zone was demonstrated by 34 of these 88 isolates, all of which except one, were isolated from halophytic plant roots (Supplementary Table 1). From these 34, 11 isolates belong to the genus *Bacillus*, another 11 to *Pseudomonas* and five to *Enterobacter* (Supplementary Table 1).

Eleven isolates were tested for inhibition against the human pathogenic fungus *Aspergillus fumigatus*. Interestingly, five isolates were able to inhibit the growth of *A. fumigatus* (Figure 2). Isolate CML04 (*Bacillus altitudinis*) isolated from *Ca. maritima* was the only isolate from leaf tissues able to show inhibitory effect, while the remaining isolates were isolated from *M. triscupidata* roots (Supplementary Table 1). Two isolates MTR17d (*Bacillus sonorensis*) and MTR17b (*Bacillus licheniformis*) showed a strong inhibition zone (Figure 2 and Supplementary Table 1).

### *In-planta* Assay for Plant Growth Promotion and Salt Tolerance

Bacterial isolates with *in vitro* 10% and 17.5% NaCl salt tolerance were selected for the *in-planta* assays to demonstrate potential plant growth promotion under “no stress.” *Arabidopsis thaliana* plants were imbued with bacterial cultures and left for 7 days for the bacteria to adapt. Then, after watering the plants for a month, fresh and dry leaf weight were calculated (Table 1). The same experiment was repeated where after the 7 day mark, the plants were watered with 10 mL of 250 mM NaCl solution every 2–3 days for 30 days.

**TABLE 1:** *In-planta* plant growth promotion and salt tolerance assays in *Arabidopsis thaliana* plants. For the salt tolerance assays plants were watered with and without NaCl solution every 2-3 days and fresh and dry plant weight was measured.

		***Plant growth promotion assay#1 (sampling at 29 days***)		***Plant growth promotion assay#2 (sampling at 34 days***)		***Salinity stress assay assay#1 (sampling at 39 days***)		***Salinity stress assay assay#2 (sampling at 39 days***)	
**Host Plant**	**Isolate**	**Fresh weight**	**Dry weight**	**Fresh weight**	**Dry weight**	**Fresh weight**	**Dry weight**	**Fresh weight**	**Dry weight**
*Cakile maritima*	CML12	0.410	0.029	1.500	0.109	0.246	0.034	0.307	0.038
*Cakile maritima*	CML15	0.204	0.025	1.240	0.135	0.309	0.052	0.347	0.060
*Cakile maritima*	CMR13	0.144	0.022	1.134	0.170	0.239	0.032	0.252	0.034
*Crithmum maritimum*	CrL01	0.541	0.053	1.761	0.168	0.147	0.031	0.186	0.041
*Crithmum maritimum*	CrL04	0.149	0.019	1.169	0.137	0.197	0.031	0.203	0.032
*Crithmum maritimum*	CrL11	0.242	0.026	1.244	0.130	0.236	0.034	0.217	0.030
*Crithmum maritimum*	CrR16	0.277	0.026	1.307	0.123	0.165	0.031	0.194	0.035
*Crithmum maritimum*	CrR22	0.094	0.016	1.003	0.150	0.275	0.042	0.297	0.045
*Crithmum maritimum*	CrR23	0.170	0.023	1.173	0.148	0.103	0.026	0.127	0.030
*Matthiola tricuspidata*	MTL01	0.113	0.017	1.132	0.145	0.202	0.037	0.213	0.038
*Matthiola tricuspidata*	MTR05	0.169	0.028	1.139	0.184	0.322	0.056	0.364	0.062
*Matthiola tricuspidata*	MTR27	0.114	0.015	1.119	0.150	0.213	0.033	0.209	0.033
	Control *E. coli*	0.110	0.017	1.111	0.171	0.235	0.034	0.216	0.031
	Control H2O	0.156	0.023	1.056	0.089	1.266	0.094	1.212	0.088
	Control Salt	*N/A*	*N/A*	*N/A*	*N/A*	0.250	0.034	0.221	0.029

Under no stress conditions, plants inoculated with isolates CML12, CML15, CrL01, CrL11, CrR16, CrR23, MTR05 showed an increase in fresh leaf weight between 1.1 and 2.6 times to the non-inoculated plants and between 1.0 and 2.3 times increase in dry leaf weight (Table 1). Under salt stress, the growth promotion effect was less accentuated, since plants imbued with isolates CML15, CrR22, MTR05 had less increased fresh and dry leaf (Table 1).

Isolates CML15 and MTR05 conferred an increase in fresh and dry leaf weight both under no stress and under salt stress whereas isolate CrR22 had a positive affect only under salt stress condition (Table 1). On the other hand, isolates CML12, CrL01, CrL11, CrR16, and CrR23 had a positive effect on fresh and dry weight under no stress condition (Table 1).

### Direct and Indirect *in vitro* Effects of *Verticillium dahliae* Growth

The selected 16 bacterial isolates with the exception of MTR17h inhibited significantly *V. dahliae* growth rate in dual-culture assays. However, only MTR17h, MTR17b, and MTR17c could suppress fungal growth by means of volatile compounds (Table 2). Likewise, nearly all isolates were capable of inhibiting fungal sporulation (except of MTR17g) in dual-culture assays. Most isolates significantly inhibited spore production in dual-plate assays. Interestingly, three isolates caused a significant induction of *V. dahliae* sporulation in such assays (MTR17h, MTR17b, and MTR17c), indicating that fungal growth suppression induces fungal sporulation (Table 2). Moreover, six out of 16 isolates could significantly reduce hyphae width in direct culture conditions, whereas seven out of 16 were capable of hyphae width reduction by the mean of volatiles. Additionally, nine isolates significantly inhibited microsclerotia formation in dual-culture assays; however, only three isolates significantly reduced microsclerotia formation in dual-plate assays (Table 2). MTR17h caused significant induction in microsclerotia formation both in dual-culture and in dual-plate assays.

**TABLE 2:** Values of fungal parameters of *Verticillium dahliae* treated with 16 different bacterial isolates (CrR14, CrR18, CrR04, MTR12, MTR18, CM0R1, CMR03, CML04, CMR25, MTR17a, MTR17d, MTR17f, MTR17g, MTR17h, MTR17b, MTR17c) and *Trichoderma harzianum* strain T22 in dual-culture and dual-plate assays. Values were estimated as the percentage of inhibition compared to control (V.d.).

**Treatment**	**Fungal parameters^a^**							
	**Dual-culture assays (confrontation test)**				**Dual-plate assays (volatile test)**			
	**RGI (%)**	**SI (%)**	**HWT (%)^b^**	**MFI (%)**	**RGI (%)^c^**	**SI (%)^d^**	**HWT (%)^e^**	**MFI (%)^f^**
V.d.	0.00 h	0.00 e	0.00 d	0.00 f	0.00 cde	0.00 b	0.00 de	0.00 cde
V.d. + CrR14	52.92 c	70.87 cd	25.21 abc	68.98 ab	8.75 c	83.08 a	18.46 abcd	13.56 bcd
V.d. + CrR18	76.78 b	57.11 d	33.53 a	52.41 bcde	−1.97 cde	79.68 a	22.33 ab	24.99 cde
V.d. + CrR04	45.73 cd	74.30 bc	20.56 abcd	47.88 bcde	4.66 cd	21.84 ab	21.09 abc	71.91 ab
V.d. + MTR12	23.97 ef	92.94 a	22.92 abc	26.65 cdef	−3.83 cde	82.75 a	25.99 a	−20.10 cde
V.d. + MTR18	33.95 de	89.36 bc	23.49 abc	34.23 bcdef	−2.78 cde	83.13 a	30.95 a	−1.86 cde
V.d. + CMR01	21.91 ef	81.46 abc	30.26 ab	60.41 abcd	−1.54 cde	84.81 a	30.72 a	−25.08 cde
V.d. + CMR03	27.22 ef	88.34 abc	17.56 abcd	47.42 bcde	7.70 c	56.63 ab	21.45 abc	42.98 abc
V.d. + CML04	59.69 c	70.31 cd	18.10 abcd	65.11 abc	−1.10 cde	78.68 a	28.22 a	−32.44 de
V.d. + CMR25	23.15 ef	79.00 abc	13.67 abcd	23.94 def	−3.58 cde	70.63 ab	17.55 abcd	−12.64 cde
V.d. + MTR17a	45.22 cd	82.66 abc	6.89 cd	22.63 def	−8.86 de	−100.42 c	−5.11 e	−43.35 cd
V.d. + MTR17d	45.85 cd	83.99 abc	5.56 ab	32.93 bcdef	3.31 cd	74.61 ab	−3.78 e	−2.18 de
V.d. + MTR17f	51.46 c	74.21 bc	10.89 bcd	18.77 ab	−16.21 e	77.33 ab	1.56 de	−60.99 e
V.d. + MTR17g	16.75 fg	5.29 e	21.33 abc	62.41 abcd	−4.50 cde	38.33 ab	3.00 cde	−2.99 cde
V.d. + MTR17h	2.86 gh	70.53 cd	5.59 cd	−67.79 g	55.99 b	−116.97 c	5.59 bcde	−298.98 f
V.d. + MTR17b	49.02 cd	81.79 abc	0.58 d	22.91 def	74.57 a	−120.55 c	0.58 de	97.83 a
V.d. + MTR17c	60.21 c	91.26 a	7.60 cd	24.02 def	69.06 ab	−88.87 c	6.35 bcde	99.28 a
V.d. + TRIANUM-P	95.79 a	79.46 abc	nm	98.91 a	ne	ne	ne	ne

*^a^Fungal parameters were calculated according to the formula: [(Vc-Vt)/Vc] × 100 where Vc = the microscopic value of V. dahliae in control and Vt = the respective value of V. dahliae toward the antagonistic isolate in dual-culture or dual-plate assays. Each value represents the mean of 3 replicates. RGI*, (*radial growth inhibition; SI*, (*sporulation (spore production) inhibition; HWT*, (*hyphae width thinning; MFI*, (*microsclerotia formation inhibition. Within columns, values followed by the same letter are not significantly different according to Tukey’s HSD test at P ≤ 0.05.*

*^b^“nm” indicates that HWT values were not measured since T. harzianum overgrown V. dahliae in dual-culture assays and pathogen hyphae could not be identified.*

*^c, d, e, f^“ne” indicates that RGI, SI, HWT and MFI were not estimated since T. harzianum could reach directly V. dahliae even in dual-plate assays.*

### Suppression of *Verticillium* Wilt Symptoms *in-planta*

For the suppression of *Verticillium dahliae* wilt symptoms *in-planta* we used a well-established fungus/plant system, the *Verticillium*/eggplant system. We selected 16 bacterial isolates that showed promising *in vitro* growth inhibition effect to *Verticillium*.

**FIGURE 3:**
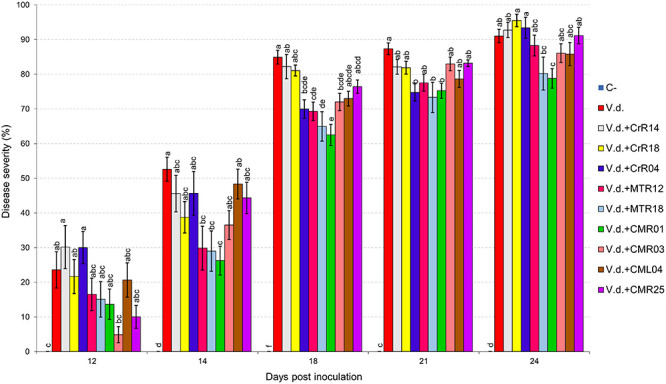
Verticillium wilt disease severity index on eggplant treated with various bacterial isolates at 12, 14, 18, 21, and 24 days post inoculation with *Verticillium dahliae* conidial suspension (20 mL of 5 × 10^6^ conidia mL^–1^). Each column represents the mean of 21 plants after combining the results of 3 replicated experiments (experiment I). Columns at each observation time point followed by the same letter are not significantly different according to Tukey’s HSD test at *P* ≤ 0.05. Vertical bars indicate standard errors.

**FIGURE 4:**
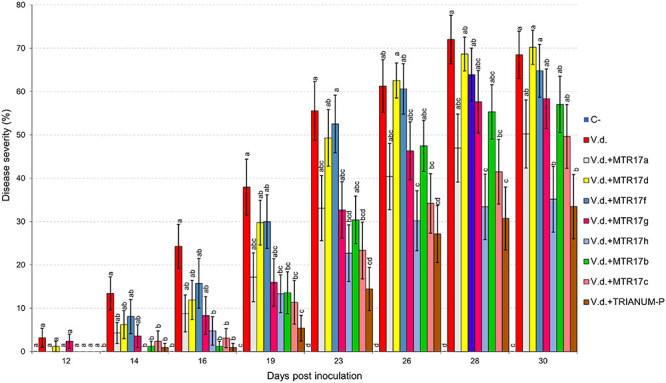
Verticillium wilt disease severity index on eggplant treated with various bacterial isolates and the commercial biofungicide TRIANUM-P (Koppert B.V. Hellas) at 12, 14, 16, 19, 23, 26, 28, and 30 days post inoculation with *Verticillium dahliae* conidial suspension (20 mL of 5 × 10^6^ conidia mL^–1^). Each column represents the mean of 21 plants after combining the results of 3 replicated experiments (experiment II). Columns at each observation time point followed by the same letter are not significantly different according to Tukey’s HSD test at *P* ≤ 0.05. Vertical bars indicate standard errors.

Two distinct assays were performed (hereafter known as “experiment I” and “experiment II”). *V. dahliae* wilt symptoms on eggplant started 12 days after inoculation (d.p.i.), with *V. dahliae* conidial suspension and were recorded periodically for another 12 days in experiment I. Isolates CrR4, MTR12, MTR18, and CMR01 suppressed significantly disease severity at 18 and 21 d.p.i. whereas MTR18 and CM1 treatments caused significant reduction of disease severity at most observation time points (Table 2 and Figures 3, 4). Considering all disease parameters, CMR01 was the most effective isolate in terms of disease suppression (Table 2, Figure 3, and Supplementary Tables 2, 3). First disease symptoms in experiment II were also observed on 12 d.p.i. and recorded until 30 d.p.i. Disease severity progressed rapidly in the control (*V.d.*) and the non-suppressive treatments (MTR17d, MTR17f, and MTR17g), whereas MTR17a-, MTR17h-, MTR17b-, and MTR17c-treated plants showed less prominent symptoms and slower disease development (Table 2 and Supplementary Figure 3). Disease parameters indicated that isolate MTR17h, is comparable to the positive control (fungus *Trichoderma harzianum* isolate + TRIANUM-P), as the most effective in symptom suppression (Table 2 and Figure 4). While observed decrease in symptom severity in MTR17h-treated plants was associated with significantly lower *V. dahliae* re-isolation ratio compared to positive control (*V.d.*) plants, MTR17h isolate did not show strong growth inhibition effect on *V. dahliae* in *in vitro* assays (Figure 4), indicating less active growth of the pathogen into the xylem vessels. This finding could suggest that the plant innate immunity activation/reinforcement effect by MTR17h, needs to be further investigated in the future. Neither symptoms nor positive isolations were observed in negative control plants.

### Effects of Treatments in Plant Growth

Growth parameters of eggplant inoculated with *V. dahliae* and treated with the 16 isolates and the *T. harzianum* isolate TRIANUM-P or not (C−), are shown on Table 3. *V. dahliae*-inoculated plants treated with MTR17c and *T. harzianum* TRIANUM-P developed significantly higher fresh weight compared with the *V. dahliae*-inoculated controls, whereas most of the plant growth parameters in non-inoculated plants were significantly higher than the inoculated ones.

**TABLE 3:** Values (± standard errors) of disease parameters for eggplants inoculated with *V. dahliae* and treated with different bacterial isolates and TRIANUM-P (CrR14, CrR18, CrR04, MTR12, MTR18, CMR01, CMR03, CML04, CMR25 in experiment I, and MTR17a, MTR17d, MTR17f, MTR17g, MTR17h, MTR17b, MTR17c, TRIANUM-P in experiment II) or not (C−, V.d.).

**Experiment**	**Treatment**	**Disease parameters^a^**				
		**DI (%)**	**FDS (%)**	**M (%)**	**RAUDPC (%)**	**IR**
	C−	0.00 ± 0.00b	0.00 ± 0.00c	0.00 ± 0.00c	0.00 ± 0.00c	0.00 ± 0.00b
	V.d.	100.00 ± 0.00a	91.00 ± 1.91*ab*	100.00 ± 0.00a	42.36 ± 2.08a	0.55 ± 0.07*ab*
	V.d. + CrR14	100.00 ± 0.00a	92.74 ± 2.21*ab*	95.24 ± 4.76a	42.54 ± 2.62a	0.65 ± 0.05a
	V.d. + CrR18	100.00 ± 0.00a	95.50 ± 1.80a	90.48 ± 6.15*ab*	39.19 ± 1.99*ab*	0.55 ± 0.08*ab*
	V.d. + CrR04	100.00 ± 0.00a	93.40 ± 2.99a	78.57 ± 7.70*ab*	39.84 ± 2.18*ab*	0.60 ± 0.05*ab*
Experiment I	V.d. + MTR12	100.00 ± 0.00a	88.29 ± 2.74*abc*	80.95 ± 9.91*ab*	33.86 ± 2.59*ab*	0.80 ± 0.07a
	V.d. + MTR18	100.00 ± 0.00a	80.19 ± 4.75*bc*	71.43 ± 8.69*ab*	31.67 ± 3.02b	0.55 ± 0.05*ab*
	V.d. + CMR01	100.00 ± 0.00a	78.79 ± 2.85c	52.38 ± 14.29b	30.72 ± 2.18b	0.55 ± 0.08*ab*
	V.d. + CMR03	100.00 ± 0.00a	86.07 ± 2.71*abc*	66.67 ± 14.55*ab*	32.25 ± 1.47b	0.65 ± 0.08a
	V.d. + CML04	100.00 ± 0.00a	85.82 ± 3.34*abc*	69.05 ± 5.67*ab*	37.91 ± 2.00*ab*	0.65 ± 0.05a
	V.d. + CMR25	100.00 ± 0.00a	91.17 ± 2.37*ab*	80.95 ± 9.91*ab*	35.71 ± 1.46*ab*	0.85 ± 0.03a

	C−	0.00 ± 0.00c	0.00 ± 0.00c	0.00 ± 0.00b	0.00 ± 0.00*d*	0.00 ± 0.00b
	V.d.	90.48 ± 6.15*ab*	68.49 ± 5.43a	47.62 ± 9.91a	26.76 ± 2.95a	0.53 ± 0.05a
	V.d. + MTR17a	71.43 ± 15.31*ab*	50.21 ± 7.88*ab*	33.33 ± 10.29*ab*	15.05 ± 2.81*bc*	0.20 ± 0.09*ab*
	V.d. + MTR17d	95.24 ± 4.76a	70.22 ± 3.93a	23.81 ± 14.02*ab*	23.04 ± 2.20*ab*	0.40 ± 0.20*ab*
Experiment I I	V.d. + MTR17f	85.71 ± 9.91*ab*	64.79 ± 6.12a	33.33 ± 14.51*ab*	22.95 ± 2.67*ab*	0.38 ± 0.17*ab*
	V.d. + MTR17g	80.95 ± 6.73*ab*	58.36 ± 6.85*ab*	38.10 ± 11.34*ab*	16.81 ± 2.84*abc*	0.27 ± 0.12*ab*
	V.d. + MTR17h	52.38 ± 12.30b	35.15 ± 7.58b	4.76 ± 4.76b	10.51 ± 2.58*cd*	0.10 ± 0.04b
	V.d. + MTR17b	80.95 ± 9.91*ab*	57.05 ± 6.52*ab*	19.05 ± 6.73*ab*	14.85 ± 2.03*bc*	0.40 ± 0.18*ab*
	V.d. + MTR17c	76.19 ± 9.52*ab*	49.66 ± 7.35*ab*	23.81 ± 6.15*ab*	11.73 ± 2.35c	0.13 ± 0.06*ab*
	V.d. + TRIANUM-P	52.38 ± 6.74b	33.45 ± 7.44b	4.76 ± 4.76b	7.88 ± 1.94*cd*	0.20 ± 0.09*ab*

**^*a*^Disease parameters were evaluated periodically on the basis of external symptoms during a period of 24 days (in Experiment I) and 30 days (in Experiment II) after root drenching with Verticillium dahliae conidial suspension (20 mL of 5 × 10^6^ conidia mL^–1^ per plant). One week prior to inoculation with V. dahliae, plants were root-drenched with bacterial suspension (20 mL of 10^8^ cfu mL^–1^ of each isolate per plant); whereas TRIANUM-P was also included in experiment II and applied by root drenching (20 mL of 3 × 10^7^ cfu mL^–1^ per plant). DI*, (*final disease incidence; FDS*, (*final disease severity; M*, (*mortality; RAUDPC*, (*relative area under the disease progress curve with reference to the maximum value potentially reached over each assessment period; IR*, (*isolation ratio. Each value represents the mean of 21 plants after combining the results of 3 replicated experiments (except from IR that represents the mean of 5 plants in total). Within experiments, values in columns followed by the same letter are not significantly different according to Tukey’s HSD test at P ≤ 0.05.**

### Whole-Genome Sequencing and Analysis of Selected Endophytic Bacterial Isolates

Whole-genome sequencing (WGS) was performed on 12 selected isolates. Genomes were annotated using RAST (Supplementary Figure 5). All genes related to the virulence, disease and defense that were predicted are presented in Table 4 and Supplementary Table 2.

**TABLE 4:** Number of genes related to Virulence, Disease and Defense features of the three new bacterial species identified in this study. The genome analysis and the annotation was performed using the RAST genome annotation software.

**Virulence, disease and defense**	***Arthrobacter* sp. CMR16**	***Pseudomonas* sp. CrR25**	***Pseudomonas* sp. CMR27**	***Pseudomonas* sp. CMR25**
**Resistance to antibiotics and toxic compounds**	**19**	**56**	**42**	**45**
Mercury resistance operon	1	0	0	0
Copper homeostasis	6	25	18	18
Cobalt-zinc-cadmium resistance	4	12	17	16
Resistance to fluoroquinolones	2	5	2	5
Copper homeostasis: copper tolerance	2	2	2	2
Beta-lactamase	1	0	2	1
Mercuric reductase	3	3	0	0
Multidrug Resistance Efflux Pumps	0	7	0	0
Resistance to chromium compounds	0	1	1	3
**Invasion and intracellular resistance**	**19**	**21**	**14**	**17**
Mycobacterium virulence operon involved in protein synthesis (SSU ribosomal proteins)	6	9	6	7
Mycobacterium virulence operon involved in DNA transcription	3	6	2	4
Mycobacterium virulence operon possibly involved in quinolinate biosynthesis	3	3	3	3
Listeria surface proteins: Internalin-like proteins	4	0	0	0
Mycobacterium virulence operon involved in protein synthesis (LSU ribosomal proteins)	3	3	3	3
**Bacteriocins, ribosomally synthesized antibacterial peptides**	**0**	**2**	**2**	**2**
Tolerance to colicin E2	0	2	2	2
**Membrane Transport**	**14**	**77**	**86**	**83**
Protein secretion system, Type II (Widespread colonization island)	11	14	10	10
Protein secretion system, Type II (General Secretion Pathway)	0	15	0	0
Protein secretion system, Type V (Two partner secretion pathway–TPS)	0	4	0	0
Protein secretion system, Type I	0	0	29	22
Protein secretion system, Type III	0	0	0	0
Protein secretion system, Type VI	0	0	0	0
Protein and nucleoprotein secretion system, Type IV (Type IV pilus)	0	28	22	20
Protein and nucleoprotein secretion system, Type IV (Conjugative transfer)	0	12	0	0
Protein secretion system, Type VII (Chaperone/Usher pathway, CU)	0	0	13	12
Twin-arginine translocation system	3	4	7	7
Protein secretion system, Type VIII (Extracellular nucleation/precipitation pathway, ENP)	0	0	5	12

**FIGURE 5:**
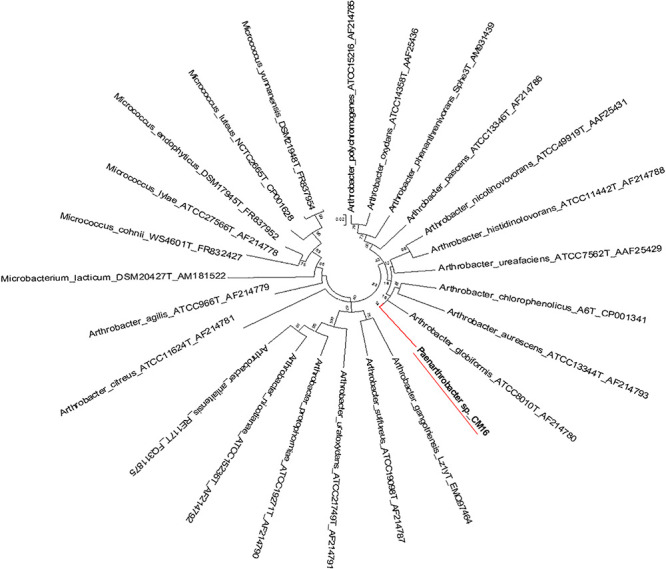
Molecular phylogenetic analysis of *Arthrobacter recA-gyrB* genes by Maximum Likelihood method. Phylogenetic tree is drawn to scale, with branch lengths measured in the number of substitutions per site.

**FIGURE 6:**
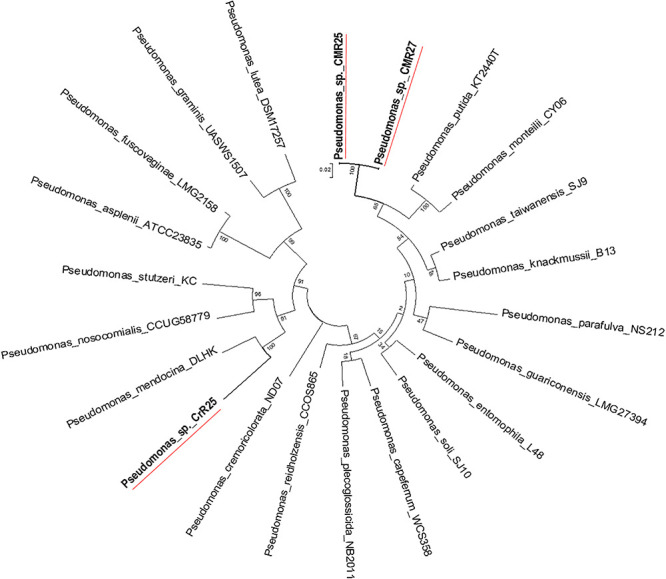
Molecular Phylogenetic analysis of the *recA-gyrB* genes from Pseudomonads belonging to *P. putida* and *P. mendocina* groups by Maximum Likelihood method. The tree with the highest log likelihood (–7831.6808) is shown. The tree is drawn to scale, with branch lengths measured in the number of substitutions per site.

Genome-wide average nucleotide identity (ANI) calculations against all bacterial genome assemblies in GenBank pointed to the existence of three previously unknown bacterial species; their genomes showed less than 95% ANI with any previously sequenced genomes. Phylogenetic analysis of the *recA* and *gyrB* gene sequences extracted from the genomes (Figures 5, 6) had indicated that two isolates belong to *Pseudomonadaceae*, while the third was a member of *Arthrobacter* genus. Isolates CMR25 and CMR27 belonged to an unidentified species of the *P. putida* group (Figure 6) and isolate CrR25 was an undefined species of the *P. mendocina* group (Figure 6). Consistent with these results, 16S rRNA gene sequences of CMR25 and CMR27 were 99.8% identical to that of *Pseudomonas plecoglossicida* and CrR25 was 98.75% identical to that of *Pseudomonas benzenivorans*. WGS analysis places CMR16 as an unidentified *Arthrobacter* species (Figure 5), while its 16S rRNA gene sequence assigns the isolate to *Paenarthrobacter nitroguajacolicus* (Kotoučková et al., 2004) with 98.78% identity. This species has been previously isolated from leaves of maize (Pisarska and Pietr, 2012) and promoted growth of wheat under salt stress (Safdarian et al., 2019). Unfortunately, no genome sequence is available for the type strain of this species; however, the ANI between CMR16 genome and previously sequenced genomes (Yao et al., 2015) of *Paenarthrobacter nitroguajacolicus* (Kotoučková et al., 2004) range between 86.08 and 86.76%, well below the widely used threshold of 96% for species membership.

## Discussion

Utilization of endophytic microorganisms for the control of biotic/abiotic stresses is a relatively unexplored area of research. Endophytes have been studied for over two decades (Saikkonen et al., 1998; Hasegawa et al., 2006; Kaul et al., 2016), however, our understanding about their role in plant defense against biotic/abiotic stresses is still limited (Liu et al., 2020; Pascale et al., 2020). Isolation, identification and the study of endophytes from plants that undergo continued abiotic stress could be essential for the development of proper biocontrol strategy for sustainable agriculture and food security.

**FIGURE 7:**
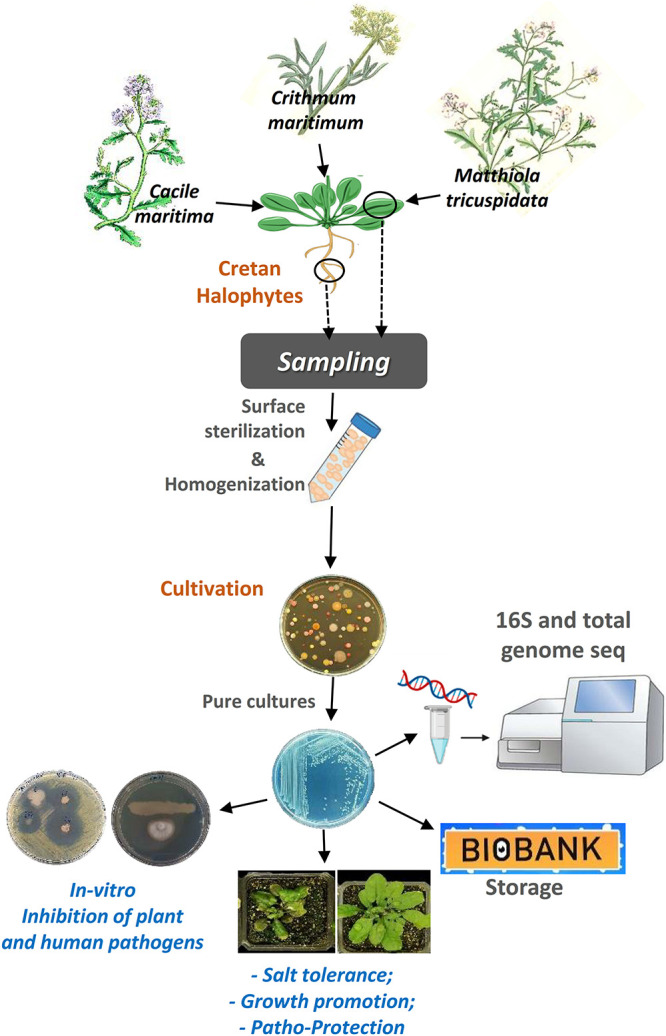
A graphical abstract describing the steps of the procedure we followed to accomplish this work.

Here, we investigated the abundance of taxa of the culturable bacterial endophytes of three halophytic plants, endemic in Crete island, Greece, using culture-dependent techniques (Figure 7). We also investigated the proof-of-concept of using the halophytes as a valuable source of beneficial microbes that can potentially be used in agriculture, by testing our initial hypothesis that these endophytes have plant growth promotion and biocontrol properties.

Taxonomically, 24 different genera were identified, the three most abundant ones were *Bacillus*, *Enterobacter*, and *Pseudomonas*, all of which have been previously observed in studies of the endophytic microbiome of halophytes (Shabala, 2013; Qin et al., 2014; Mora-Ruiz et al., 2016; Yuan et al., 2016).

*In-planta* testing of *Oceanobacillus picturae* (CML15), *Terribacillus saccharophilus* (MTR05), and *Bacillus haikouensis* (CrR22) demonstrated an increase in both dry and fresh leaf weight in *Arabidopsis thaliana* plant under salinity stress. These isolates are promising biofertilizers, since other isolates of the same species have also been shown to have plant growth promotion properties; *Terribacillus saccharophilus*, firstly reported at 2007, is a known halophilic bacterium able to grow on 0–16% NaCl (An et al., 2007; Liu et al., 2010). This species is a known endophytic bacterium (Han et al., 2011), shown to trigger an increase on monoterpenes, sesquiterpenes, tocopherols, and membrane sterols, compounds engaged in antioxidant capacity in leaf tissues of grape resulting in stress tolerance (Salomon et al., 2016). *Bacillus haikouensis* is halotolerant bacterium isolated from paddy soil, able to grow on up to 17% NaCl (Li et al., 2014). *Oceanobacillus picturae* is a halophilic phosphate-solubilizing species with demonstrated siderophore production potential, isolated from saline environments and shown to promote plant growth in mangroves and confer salinity stress tolerance in barley (El-Tarabily and Youssef, 2010; Mapelli et al., 2013; Orhan and Demirci, 2020). Many of our isolates were able to grow at high concentrations of salt (5–17% NaCl).

Isolates belonging to the species *Bacillus licheniformis, Bacillus sonorensis, Pseudomonas glareae, Enterobacter hormaechei, Pseudomonas benzenivorans, Pseudomonas monteilii, Pseudomonas plecoglossicida* were shown to have strong antagonistic activity against the phytopathogenic bacteria *Ralstonia solanacearum* and *Clavibacter michiganensis* subsp. *michiganensis*, two very important plant pathogens with high economic impact on agriculture (Gartemann et al., 2003; Peeters et al., 2013). Both are very important phytopathogens, since *Ralstonia* has a large host range able to infect more than 200 plant species easily adaptable in varying environmental conditions whereas *C. michiganensis* subsp. *michiganensis* is able to infect wheat, maize, potatoes, and red and green peppers, despite its main host being tomatoes (Eichenlaub and Gartemann, 2011; Peeters et al., 2013; Hwang et al., 2018). Moreover, specific isolates with *in vitro* growth inhibition effect against *V. dahliae*, were tested for their ability to inhibit *V. dahliae in-planta*. Several isolates demonstrated an *in-planta* suppression effect of the polyphagous pathogen *V. dahliae*. Interestingly, isolates with strong *in vitro* effect did not manage to inhibit *V. dahliae in-planta*, but other isolates with medium or low *in vitro* effect inhibited *in-planta V. dahliae* growth strongly. These data provide the proof of concept for our study but also indicate that in future studies all resulting isolates need to be investigated for their *in-planta* antifungal and/or antibacterial growth inhibition capacity.

Furthermore, the whole-genome sequencing (WGS) of selected isolates revealed three new previously unidentified bacterial species. The identification of three new species in a very small number isolates indicates the high potential of the wild halophytic endophytome in terms of identifying new microbial species with novel capabilities, that could be beneficial for both agriculture (stress tolerance, growth promotion, etc.) and potentially in clinical practice (identification of new antibiotics, antifungal compounds, etc.).

The results from the study of the microbial collection we generated, could be the basis for the future development of various synthetic “bio-inoculants,” as the isolates possess all of the following attributes for such usage: (a) they are not pathogenic and do not induce plant disease; (b) are able to colonize plants, and (c) are culturable, so they can be used in modern agriculture. Furthermore, these isolates can be the basis for future studies, including the investigation of the colonization strategies that these microbes use, as well as, the elucidation of the molecular dialogs that take place during host-root colonization; the growth promotion; the salt tolerance and the immunity activation, by unique beneficial endophytes or artificial endophytic communities.

## Data Availability Statement

The datasets presented in this study can be found in online repositories. The names of the repository/repositories and accession number(s) can be found in the article/Supplementary Material.

## Author Contributions

PS designed the research. CC, GD, AC, EM, AS, GM, and GR performed the research. CC, IL, DS, VC, EM, and PS analyzed the data. CC, EM, DS, VC, and PS wrote the manuscript. All authors have read and approved the manuscript.

## Conflict of Interest

The authors declare that the research was conducted in the absence of any commercial or financial relationships that could be construed as a potential conflict of interest.
