# Supplementary material for: Endophytic Bacterial Isolates From Halophytes Demonstrate Phytopathogen Biocontrol and Plant Growth Promotion Under High Salinity
Source: Front Microbiol. 2021 May 4;12:681567. doi: 10.3389/fmicb.2021.681567 (PMC8129196; doi:10.3389/fmicb.2021.681567)

Culturable halophytic bacterial endophytome: a potential source of beneficial microbes for a sustainable agriculture

Christos A. Christakis[1], *Georgia Daskalogiannis*[2], *Anastasia Chatzaki*[3], *Emmanouil A. Markakis*[3], *Glykeria Mermigka*[1], *Angeliki Sagia*[2], *Giulio Flavio Rizzo*[4], *Vittoria Catara*[4], *Ilias Lagkouvardos*[5], *David J. Studholme*[6], *Panagiotis F. Sarris*[1,2,6*]

[1] Institute of Molecular Biology and Biotechnology, Foundation for Research and Technology - Hellas, Heraklion, Crete, Greece.

[2] Department of Biology, University of Crete, Voutes University Campus, Heraklion, Crete, Greece.

[3] Laboratory of Plant Pathology, Department of Viticulture, Vegetable Crops, Floriculture and Plant Protection, Hellenic Agricultural Organization DIMITRA, Heraklion, Crete, Greece.

[4] Dipartimento di Agricoltura, Alimentazione e Ambiente, Università degli studi di Catania, Catania, Italy.

[5] ZIEL - Institute for Food & Health, Technical University of Munich, Freising, Germany.

[6] Biosciences, University of Exeter, Exeter, Devon, United Kingdom.

Supplementary Table S1. Bacterial isolates from leaves and roots of *Matthiola tricuspidata*, *Crithmum maritimuma* and *Cakile maritima* plants and i*n vitro* salinity assays for salinity stress, phytopathogen growth inhibition and human pathogen growth inhibition.

Supplementary_Table_S1_In_vitro_assays.xlsx

Supplementary Table S2. Analysis of variance for disease incidence (DI), final disease severity (FDS), mortality (M), relative area under disease progress curve (RAUDPC) and pathogen isolation ration (IR) for eggplants artificially inoculated with V. dahliae and treated with various bacterial strains and TRIANUM-P (CrR14, CrR18, CrR4, MTR12, MTR18, CM1, CM3, CM4, CM25 in *experiment I,* and MTR17a, MTR17d, MTR17f, MTR17g, MTR17h and MTR17b, MTR17c, TRIANUM-P in *experiment II*) or not (C-, V.d.).

		***F* values**[a]				
		**Experiment I**				
**Source**	**df**[b]	**DI**	**FDS**	**M**	**RAUDPC**	**IR**
Replication	2	0.57	0.02	0.50	0.67	1.82
Treatment	10	362.38***	96.82***	7.65***	31.57***	2.84*
Replication × Treatment	20	0.57	1.10	0.42	1.23	0.92
		**Experiment II**				
**Source**	**df**[b]	**DI**	**FDS**	**M**	**RAUDPC**	**IR**
Replication	2	0.72	0.52	0.74	1.77	0.12
Treatment	10	9.58***	12.05***	3.27**	11.57***	2.83*
Replication × Treatment	20	1.13	1.09	1.20	1.19	0.71

[a] Symbols '*' and '***' indicate significance at $P \leq 0.05$ and 0.001 levels, respectively, according to the *F* test

[b] degrees of freedom between groups

Supplementary Table S3. Analysis of variance for plant height, plant fresh weight and total number of leaves for eggplant artificially inoculated with *V. dahliae* and treated with various bacterial strains and TRIANUM-P (CrR14, CrR18, CrR4, MTR12, MTR18, CM1, CM3, CM4, CM25 in experiment I, and MTR17a, MTR17d, MTR17f, MTR17g, MTR17h, MTR17b, MTR17c, TRIANUM-P in *experiment II*) or not (C-, V.d.).

		***F* values**[a]		
		**Experiment I**		
**Source**	**df**[b]	**Height**	**Weight**	**Leaves**
Replication	2	2.43	1.12	1.65
Treatment	10	17.09***	111.03***	12.23***
Replication × Treatment	20	1.44	0.47	1.34
		**Experiment II**		
**Source**	**df**[b]	**Height**	**Weight**	**Leaves**
Replication	2	1.58	1.05	0.56
Treatment	10	3.26***	12.60***	1.42
Replication × Treatment	20	1.22	1.21	0.87

[a] Symbol '***' indicates significance at $P \leq$ 0.05 and 0.001 levels, respectively, according to the *F* test

[b] degrees of freedom between groups.

Supplementary Table S4. Bacterial genome features in the categories of virulence, disease and defence for bacterial isolates CML04, CrR16, CrR07, CMR29, CrR06, CrR14 and CrR18.

**Virulence, Disease and Defense**	***Bacillus altitudinis* CML04**	***Bacillus haikouensis* CrR16**	***Enterobacter hormaechei* CrR07**	***Enterobacter* sp. CMR29**	***Glutamicibacter halophytocola* CrR06**	***Pseudomonas seleniipraecipitans* CrR14**	***Sphingobacterium shayense* CrR18**
**Adhesion**	**0**	**0**	**4**	**5**	**0**	**0**	**1**
Accessory colonization factor	0	0	0	0	0	0	1
Mediator of hyperadherence YidE in Enterobacteria and its conserved region	0	0	4	5	0	0	0
**Bacteriocins, ribosomally synthesized antibacterial peptides**	**10**	**10**	**0**	**0**	**0**	**0**	**0**
Bacitracin Stress Response	10	10	0	0	0	0	0
**Resistance to antibiotics and toxic compounds**	**23**	**37**	**30**	**27**	**15**	**15**	**25**
MexC-MexD-OprJ Multidrug Efflux System	0	0	0	0	0	1	0
Streptothricin resistance	1	0	1	1	0	0	0
Fosfomycin resistance	0	1	1	3	0	0	0
Mercury reductase	0	0	0		2	0	0
Copper homeostasis	9	9	11	8	3	2	10
Cobalt-zinc-cadmium resistance	6	11	4	2	4	5	4
Resistance to fluoroquinolones	3	3	2	3	0	1	2
Aminoglycoside adenylyltransferases	1	0	0		0	0	0
Copper homeostasis: copper tolerance	1	1	9	10	0	2	1

Beta-lactamase	0	5	1	1	1	1	3
Mercuric reductase	1	0	0		0	0	2
Mercury resistance operon	0	0	0	0	0	0	1
Multidrug Resistance Efflux Pumps	2	5	0		2	2	0
Adaptation to d-cysteine	0	0	1	1	0	0	0
Resistance to chromium compounds	0	1	0		1	1	1
Bile hydrolysis	0	0	0	0	0	0	1
**Invasion and intracellular resistance**	**25**	**15**	**16**	**18**	**13**	**17**	**14**
Mycobacterium virulence operon involved in protein synthesis (SSU ribosomal proteins)	9	5	7	9	4	9	6
Mycobacterium virulence operon involved in DNA transcription	9	4	3	2	3	2	2
Mycobacterium virulence operon possibly involved in quinolinate biosynthesis	3	3	3	4	3	3	3
Mycobacterium virulence operon involved in protein synthesis (LSU ribosomal proteins)	4	3	3	3	3	3	3
**Bacteriocins, ribosomally synthesized antibacterial peptides**	**0**	**0**	**1**	**1**	**0**	**1**	**1**
Tolerance to colicin E2	0	0	1	1	0	1	1
**Membrane Transport**	**0**	**13**	**49**	**50**	**6**	**30**	**3**
Protein secretion system, Type II (Widespread colonization island)	0	0	0	0	3	0	0

Protein secretion system, Type II (General Secretion Pathway)	0	0	0	0	0	0	0
Protein secretion system, Type V (Two partner secretion pathway - TPS)	0	0	0	0	0	0	0
Protein secretion system, Type I	0	0	11	10	0	0	0
IProtein secretion system, Type III	0	0	0	0	0	0	0
Protein secretion system, Type VI	0	0	0	0	0	0	0
Protein and nucleoprotein secretion system, Type IV (Type IV pilus)	0	11	15	17	0	24	0
Protein and nucleoprotein secretion system, Type IV (Conjugative transfer)	0	0	0	0	0	0	0
Protein secretion system, Type VII (Chaperone/Usher pathway, CU; sigma-Fimbriae)	0	0	5	5	0	0	0
Protein secretion system, Type VII (Type 1 pili_mannose-sensitive fimbriae, gamma-fimbriae)	0	0	5	6	0	0	0
Twin-arginine translocation system	/	2	5	5	3	6	3
Protein secretion system, Type VIII (Extracellular nucleation/precipitation pathway, ENP)	0	0	8	7	0	0	0

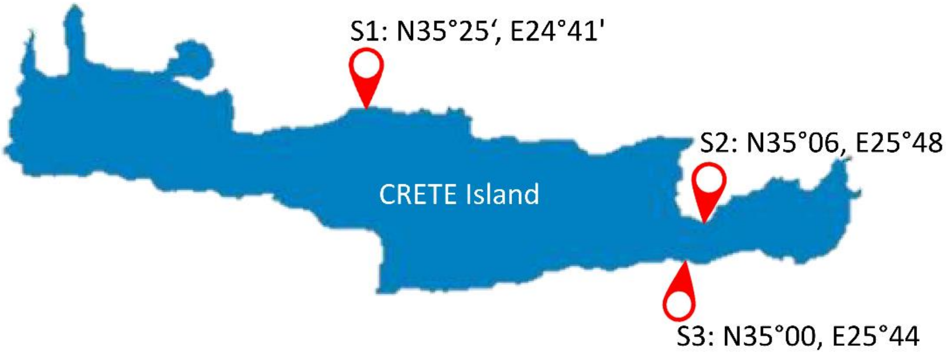

Supplementary Figure S1. Collection points and coordinates of plant samples on Crete island.

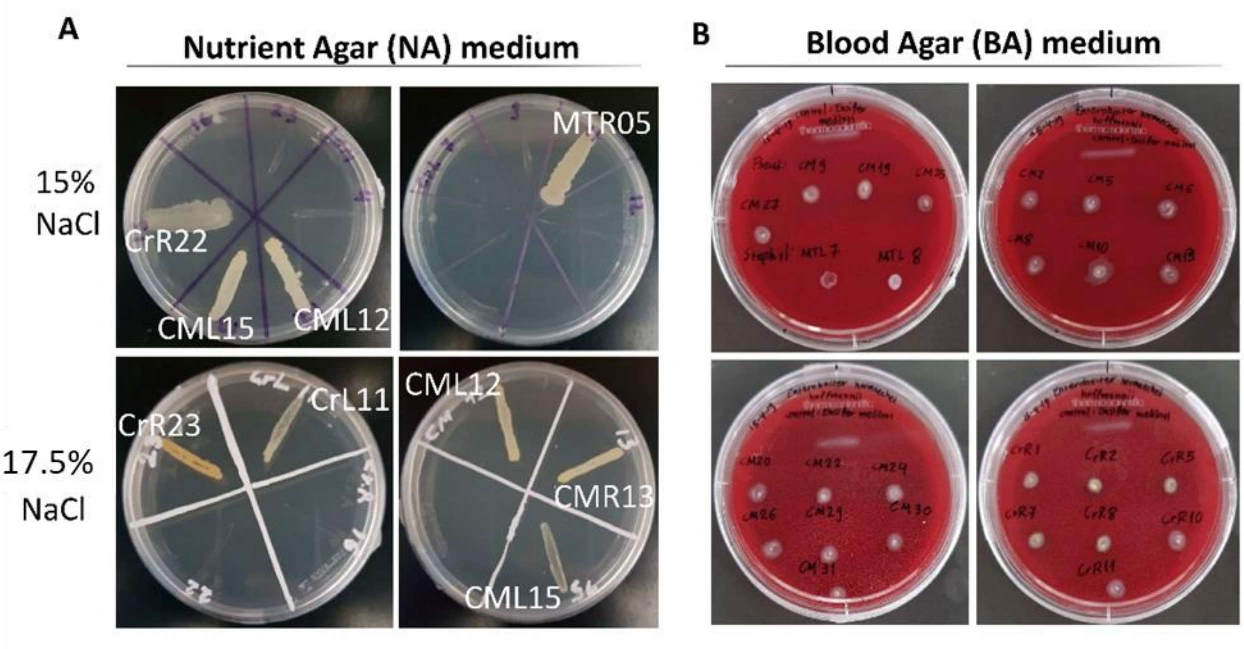

Supplementary Figure S2. A. Representative halophilic bacterial isolates cultivated in 15% and 17.5% NaCl. **B.** Haemolysis assay for the isolates that belong to opportunistic human pathogen species. Strains checked for haemolysis: CrR01; CrR02; CrR05; CrR07; CrR08; CrR10; CrR11; CMR02; CMR05; CMR06; CMR08; CMR10; CMR13; CMR20; CMR22; CMR24; CMR26; CMR29; CMR30; CMR31; CMR09; CMR19; CMR25; CMR27; MTL07; MTL08.

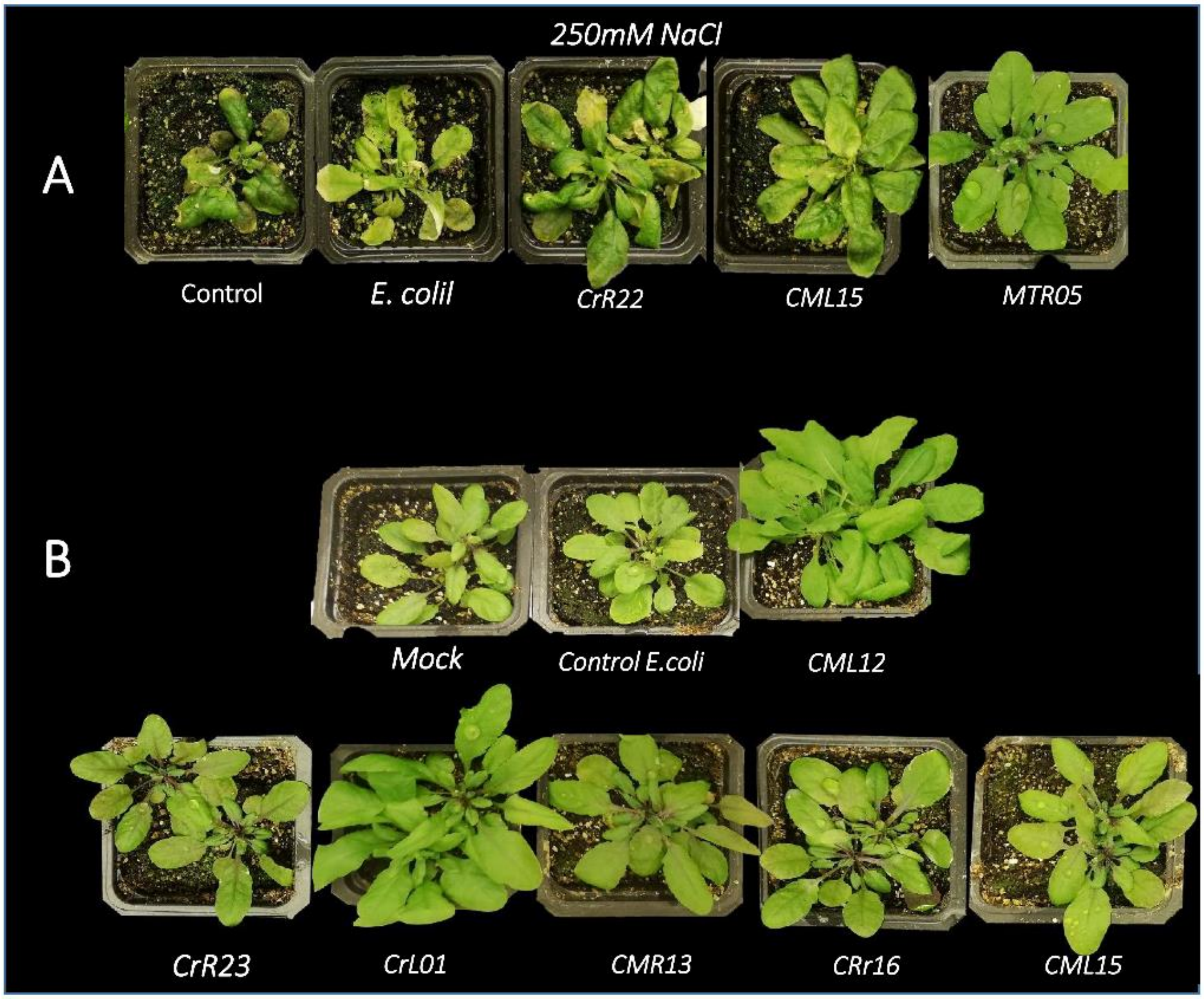

Supplementary Figure S3. Representative plants of the salt tolerance assays (**A**) and the plant growth promoting assays (**B**).

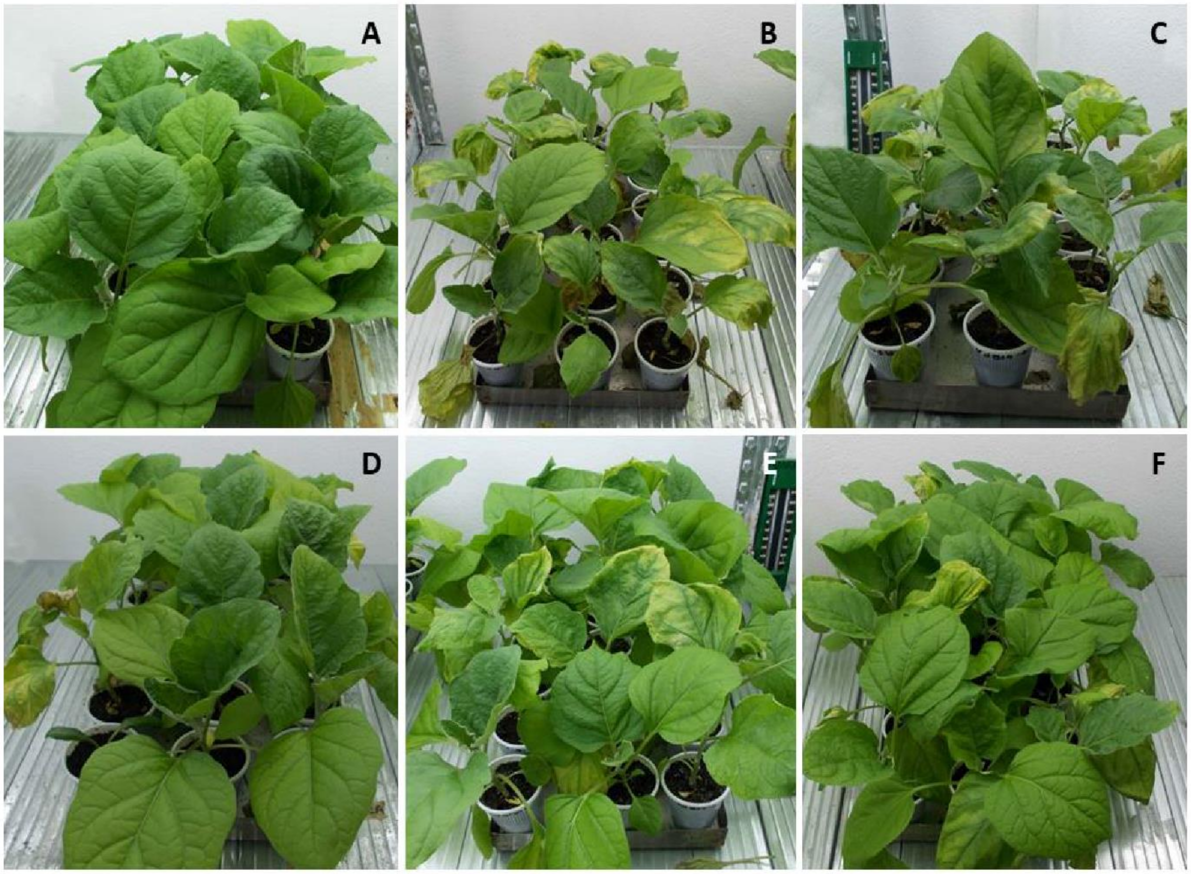

Figure S4. *Verticillium* wilt symptoms on eggplants treated with various bacterial strains and the commercial biofungicide TRIANUM-P (Koppert B.V. Hellas). **A**: mock inoculated plants treated with water (negative controls); **B**: *Verticillium dahliae*-inoculated plants with no other treatment (positive controls), **C**: V. dahliae-inoculated plants treated with the non-suppressive bacterial strain Mtr17d; **D** and **E**: V. dahliae-inoculated plants treated with the disease-suppressive strains Mtr17h and Mtr17c, respectively; **F**: *V. dahliae*-inoculated plants treated with the disease-suppressive biofungicide TRIANUM-P.

Supplementary Figure S5. Subsystem feature counts, category distribution and subsystem coverage of the gene content of fully sequenced bacterial isolates.

Pseudomonas seleniipraecipitans **CrR14**

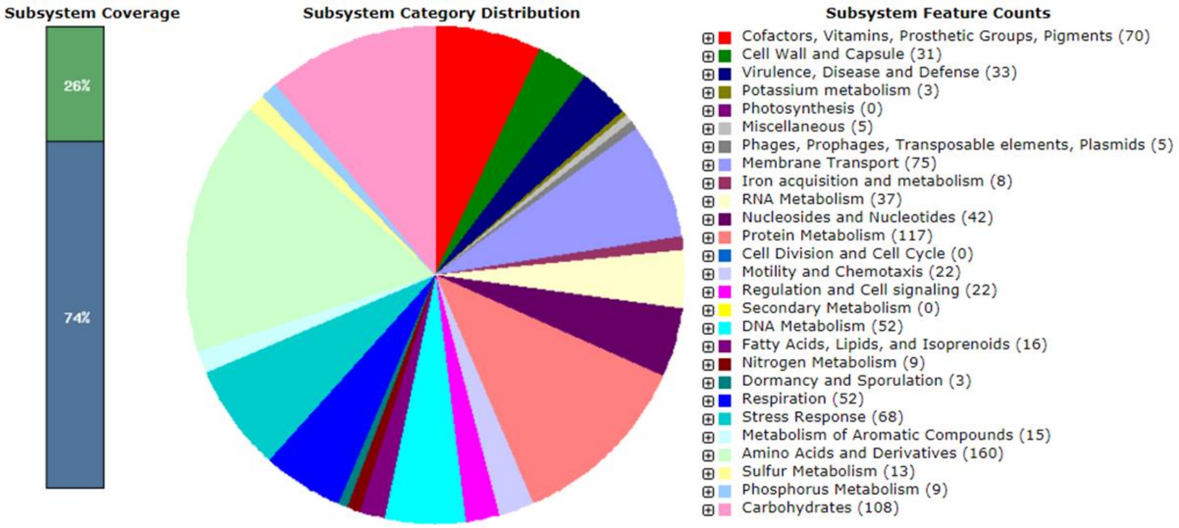

Bacillus altitudinis **CML04**

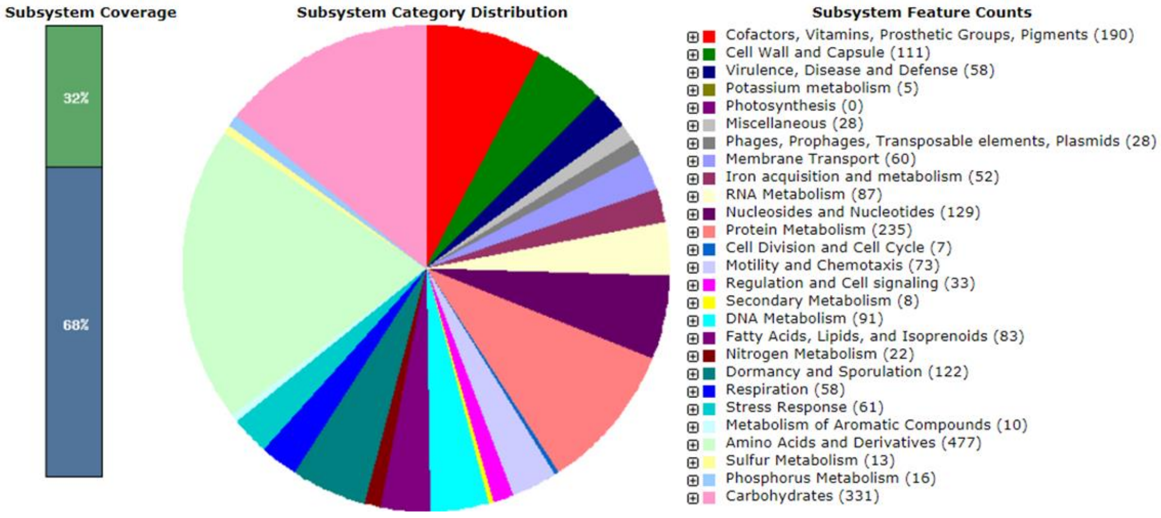

Bacillus haikouensis CrR16

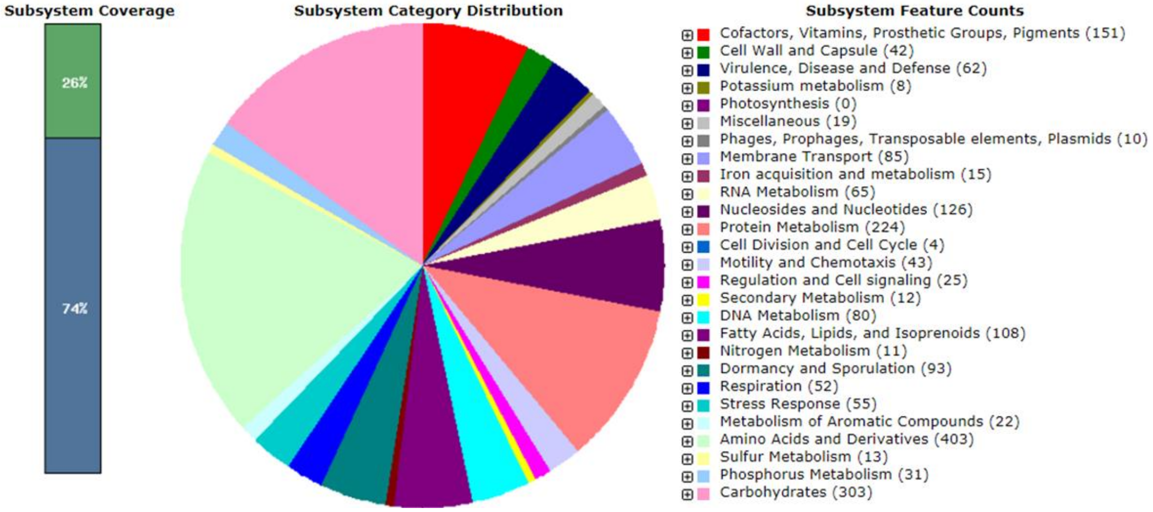

Enterobacter hormaechei CrR07

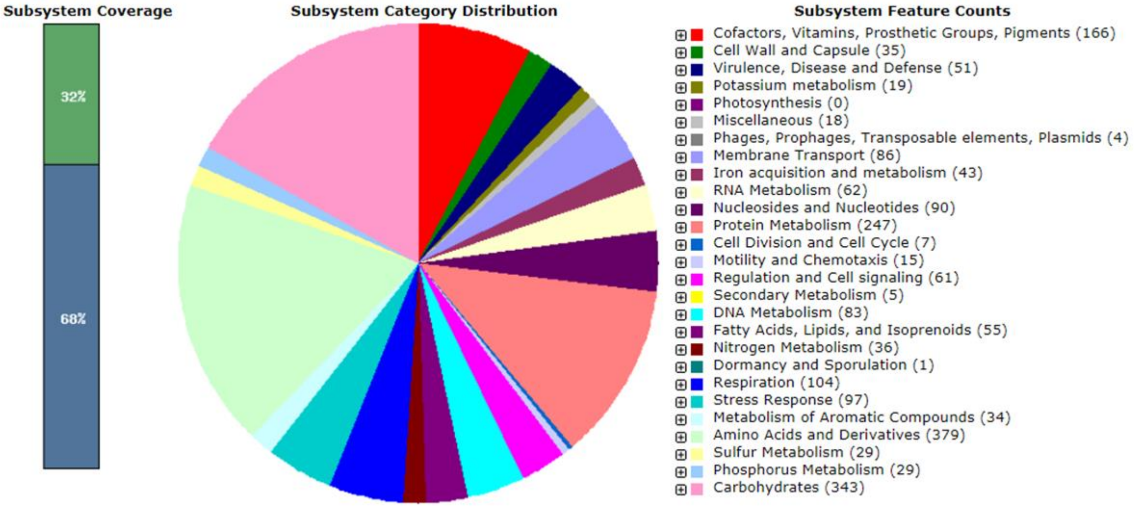

Enterobacter sp. CMR29

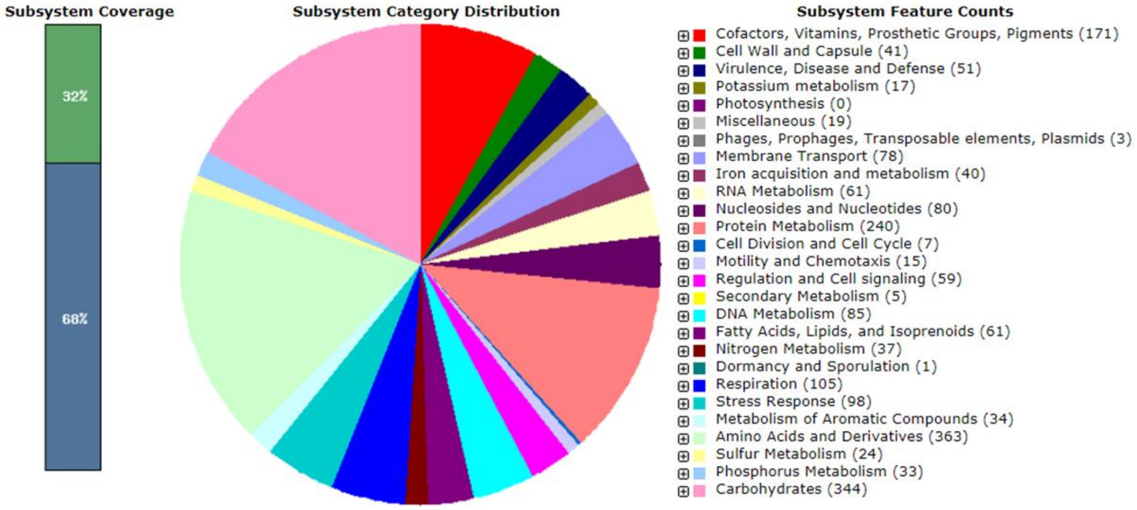

Glutamicibacter halophytocola CrR06

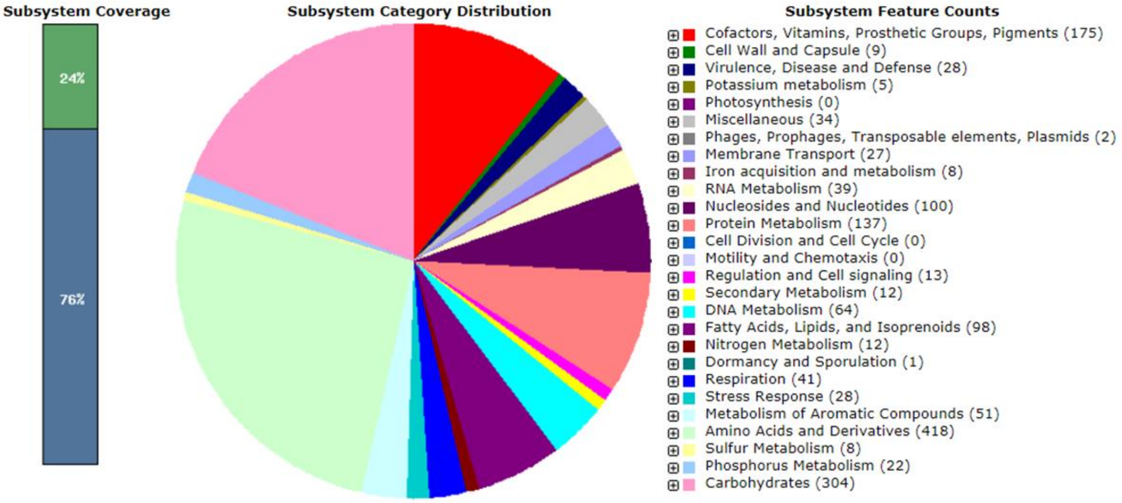

***Sphingobacterium shayense* CrR18**

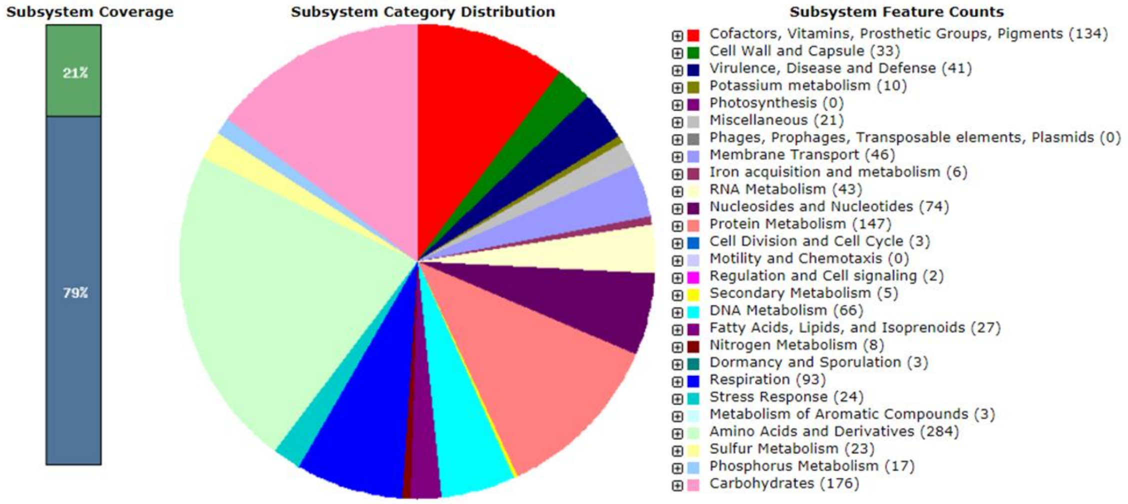

***Arthrobacter* sp. CMR16**

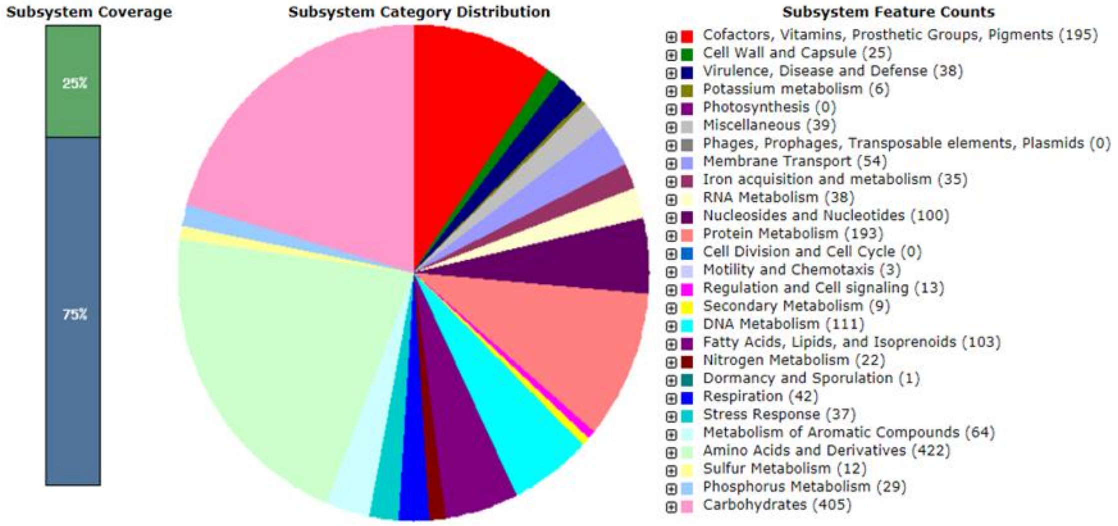

Pseudomonas sp. CrR25

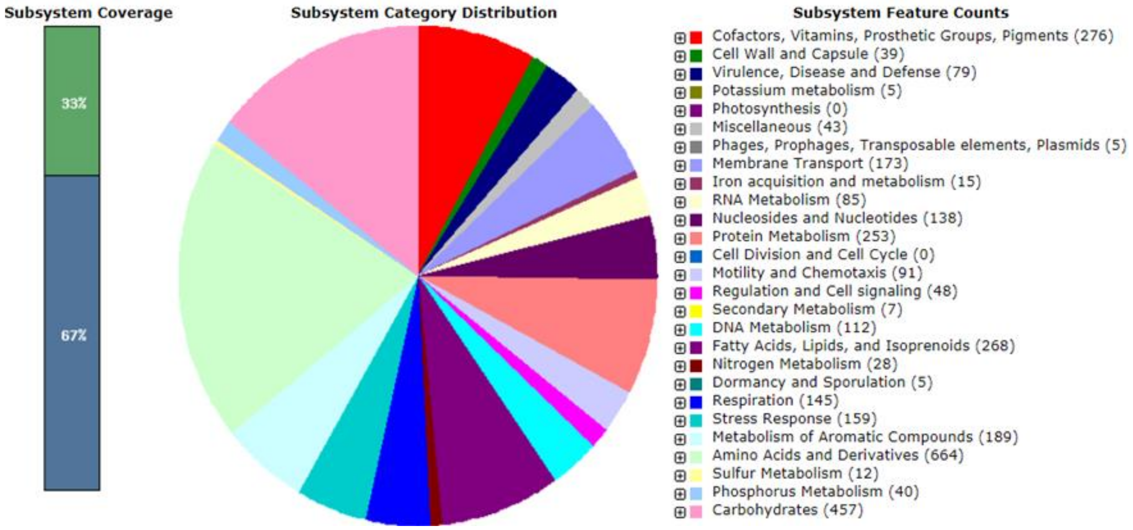

Pseudomonas sp. CMR27

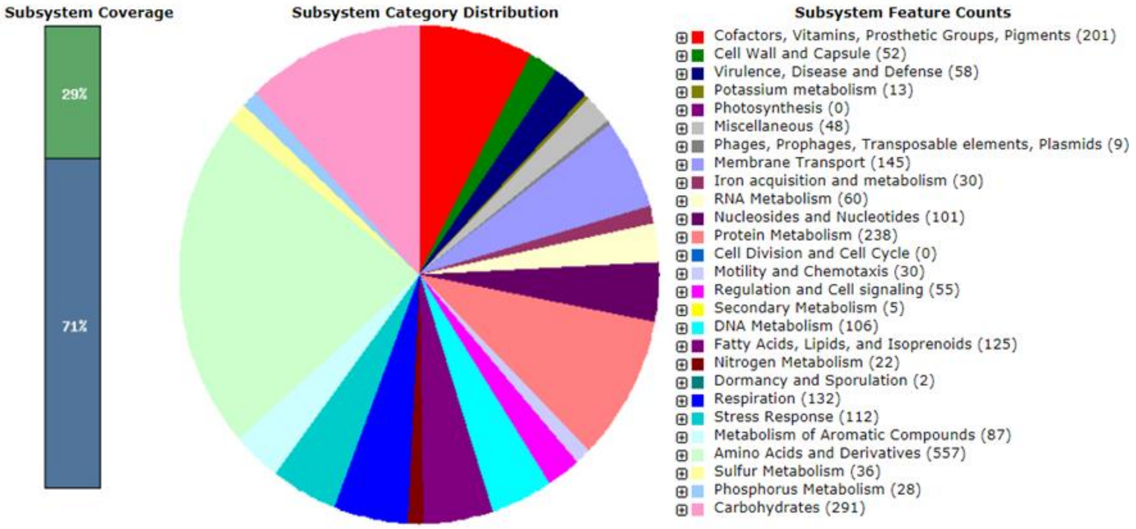

***Pseudomonas* sp. CMR25**

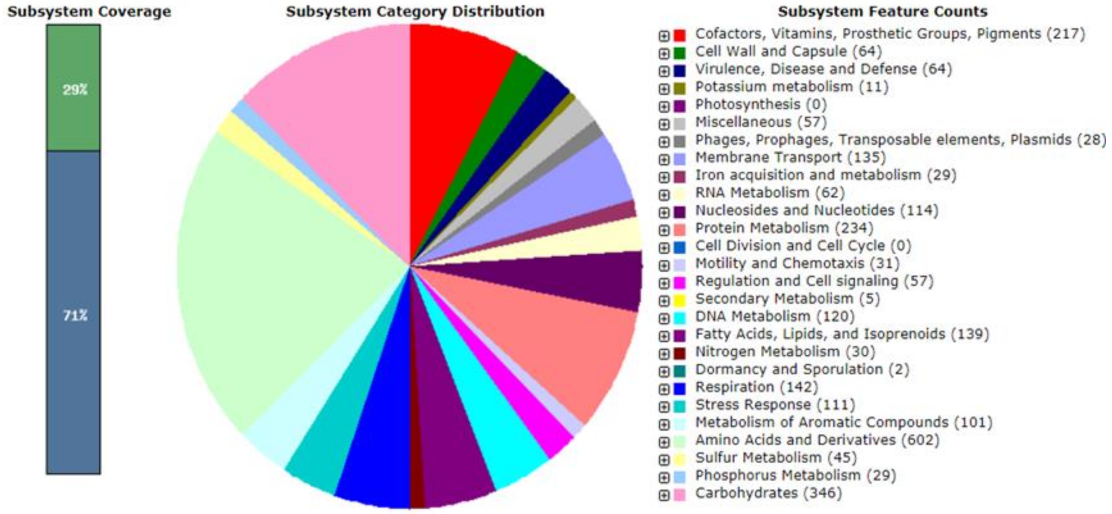

Supplement: Supplementary file 2 [file Data_Sheet_1.pdf]
